# Quality Control of ER Membrane Proteins by the RNF185/Membralin Ubiquitin Ligase Complex

**DOI:** 10.1016/j.molcel.2020.09.023

**Published:** 2020-10-15

**Authors:** Michael L. van de Weijer, Logesvaran Krshnan, Sabrina Liberatori, Elena Navarro Guerrero, Jacob Robson-Tull, Lilli Hahn, Robert Jan Lebbink, Emmanuel J.H.J. Wiertz, Roman Fischer, Daniel Ebner, Pedro Carvalho

(Molecular Cell *79*, 768–781.e1–e7; September 3, 2020)

The authors discovered two labeling errors in Figures 3C and 4A of this article. In Figure 3C, the symbols in the key were incorrect. In Figure 4A, the X and Y axes were swapped. Corrected figures appear below and with the article online. The authors apologize for the errors.

**Figure 3C:**
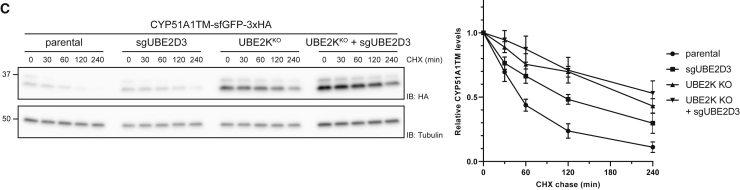
CYP51A1TM Ubiquitination and Degradation Are Dependent on RNF185 and Membralin (corrected)

**Figure 3C:**
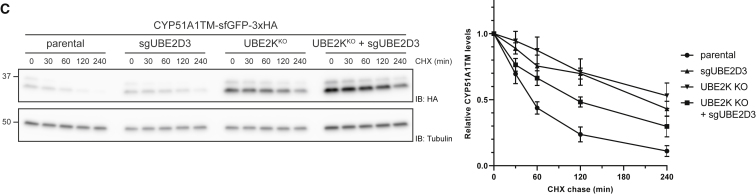
CYP51A1TM Ubiquitination and Degradation Are Dependent on RNF185 and Membralin (original)

**Figure 4A:**
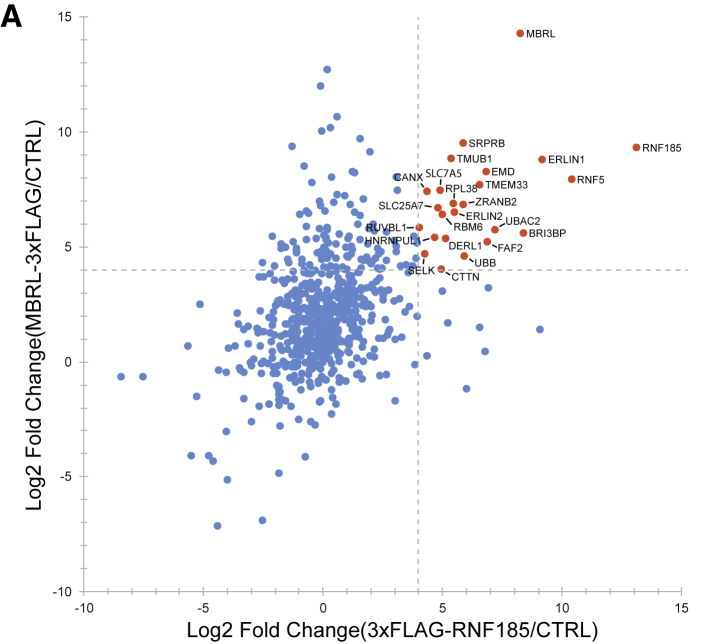
RNF185 and Membralin Form a Novel ERAD Complex (corrected)

**Figure 4A:**
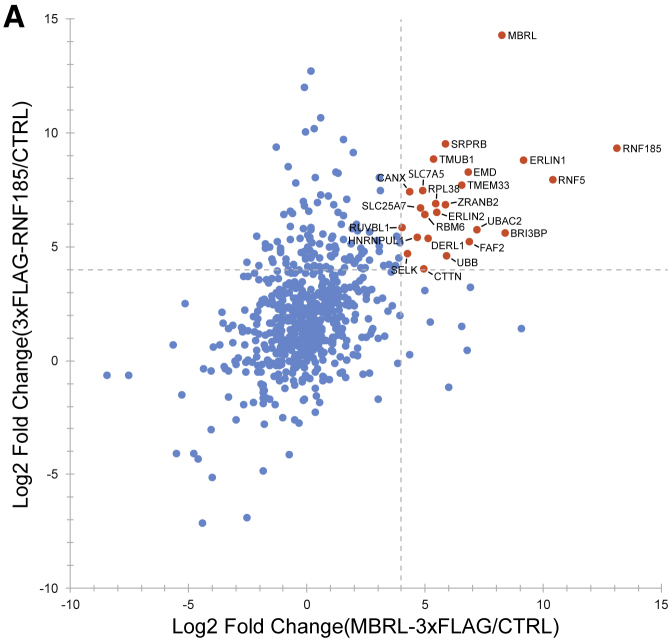
RNF185 and Membralin Form a Novel ERAD Complex (original)

