# Supplementary material for: Quality Control of ER Membrane Proteins by the RNF185/Membralin Ubiquitin Ligase Complex
Source: Mol Cell. 2020 Sep 3;79(5):768–781.e7. doi: 10.1016/j.molcel.2020.07.009 (PMC7482433; doi:10.1016/j.molcel.2020.07.009)
Supplement: Document S1. Figures S1–S6 [file mmc1.pdf]

Molecular Cell, Volume *79*

Supplemental Information

Quality Control of ER Membrane Proteins

by the RNF185/Membralin Ubiquitin Ligase Complex

Michael L. van de Weijer, Logesvaran Krshnan, Sabrina Liberatori, Elena Navarro Guerrero, Jacob Robson-Tull, Lilli Hahn, Robert Jan Lebbink, Emmanuel J.H.J. Wiertz, Roman Fischer, Daniel Ebner, and Pedro Carvalho

Supplemental Figure S1

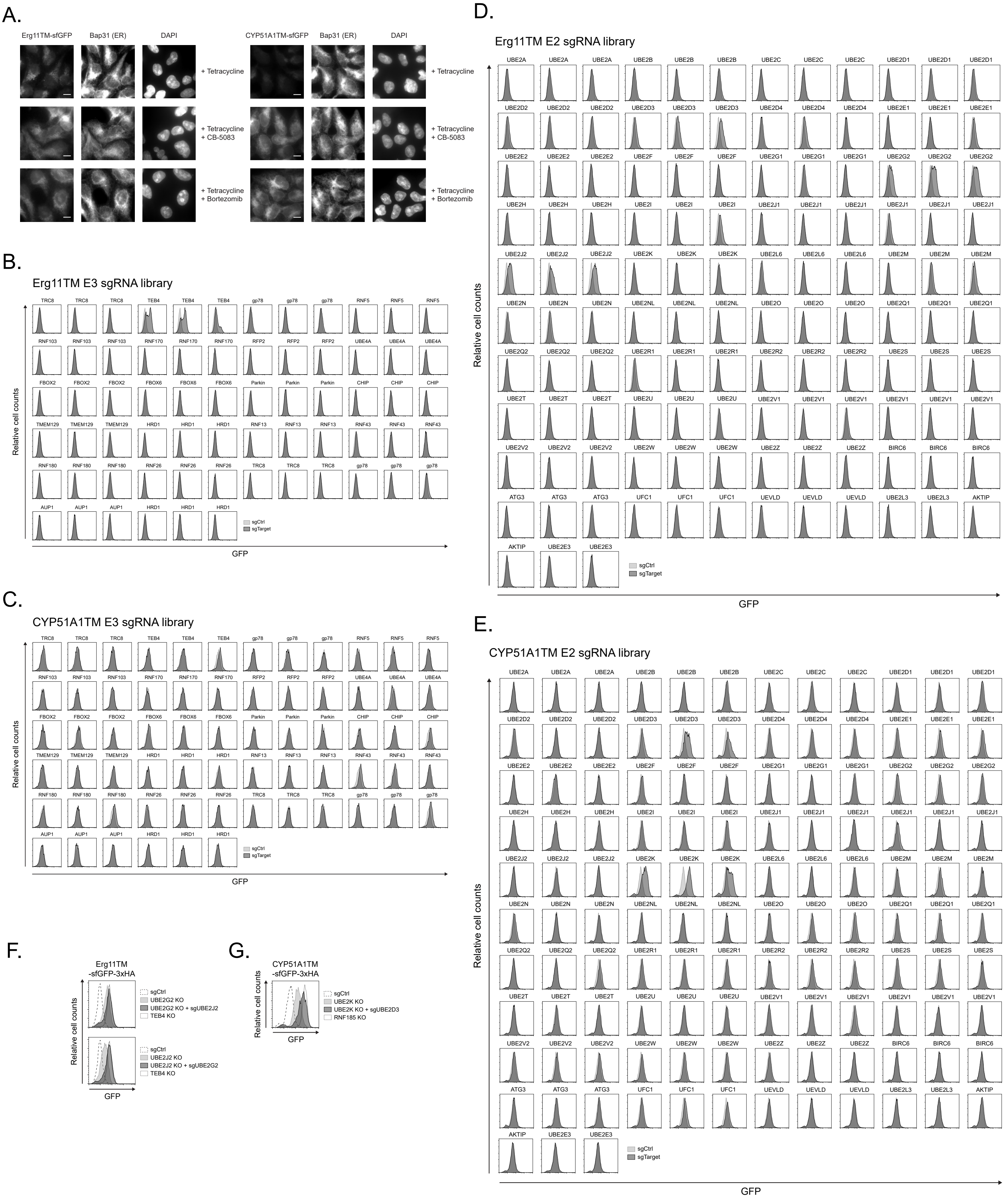

Figure S1. Focused CRISPR-Cas9 E3 and E2 library screens identify factors involved in the degradation of Erg11TM and CYP51A1TM, related to Figure 1.

(A) Erg11TM and CYP51A1TM localize to the ER. Flp-In T-REx 293 cells expressing Erg11TM or CYP51A1TM were incubated with either the p97 inhibitor CB-5083 (4 h; 2.5 μM) or the proteasome inhibitor bortezomib (4 h; 500 nM). Erg11TM and CYP51A1TM were detected by GFP fluorescence. BAP31 was used as an ER-marker and nuclei were stained with DAPI. Bars, 10 μm.

(B-E) CRISPR-Cas9 targeted screens of selected E3 and E2 libraries to identify factors involved in the degradation of Erg11TM and CYP51A1TM. Flp-In T-REx 293 cells expressing Erg11TM or CYP51A1TM were transfected with sgRNAs against indicated genes. Levels of Erg11TM and CYP51A1TM were analyzed by flow cytometry (based on GFP fluorescence).

(F-G) Redundancy between the E2 ubiquitin conjugating enzymes involved in the degradation of Erg11TM and CYP51A1TM. Flp-In T-REx 293 cells expressing (F) Erg11TM or (G) CYP51A1TM and deficient for a single E2 were additionally depleted for the indicated E2 enzyme. As a reference control, a cell line deficient for the E3 essential for the degradation of either Erg11TM or CYP51A1TM was included. Levels of Erg11TM and CYP51A1TM were analyzed by flow cytometry (based on GFP fluorescence).

Supplemental Figure S2

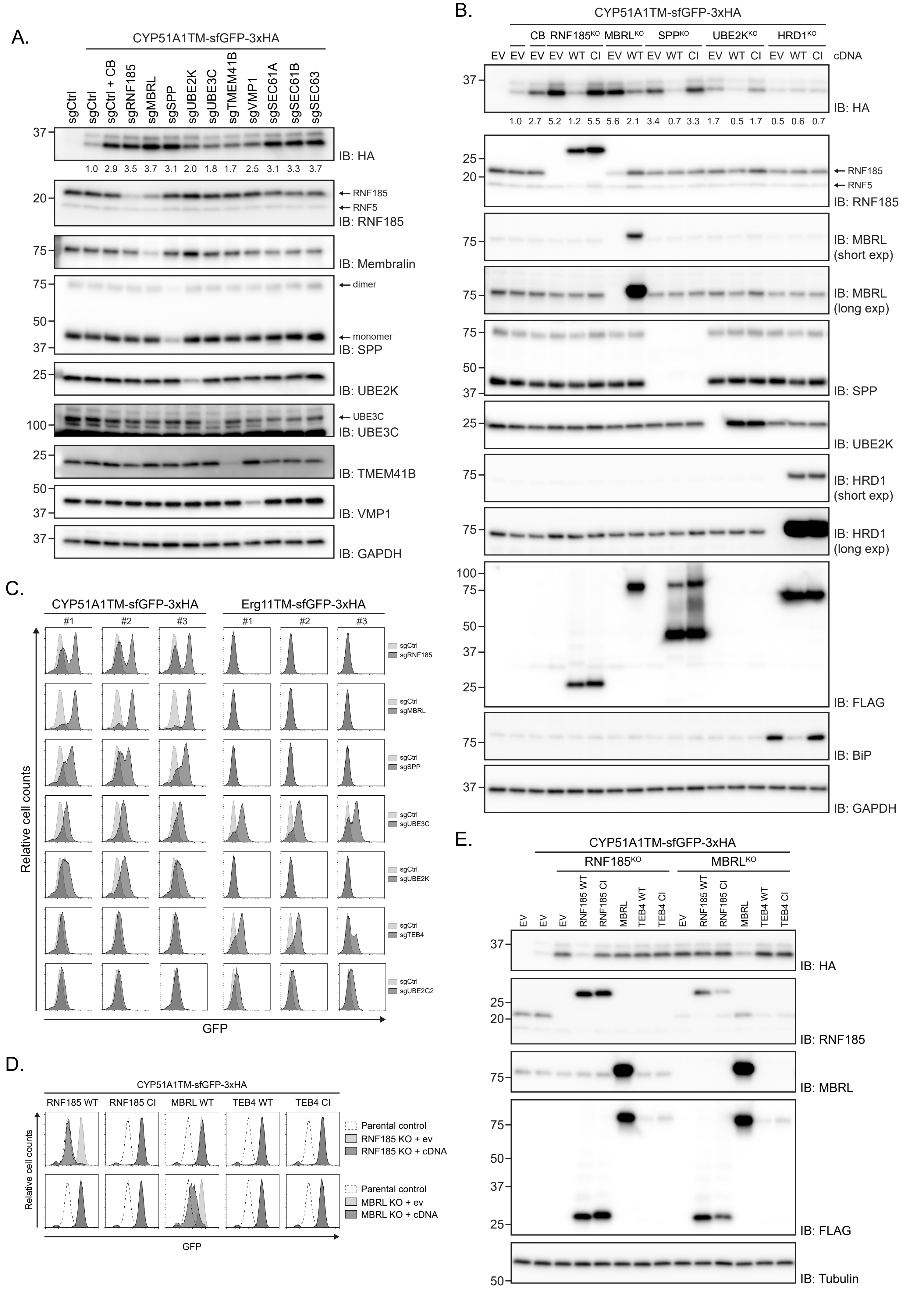

Figure S2: Additional validation of the hits identified in the CRISPR-Cas9 screen, related to Figure 2.

(A) Lysates of cells transfected with indicated sgRNAs were subjected to SDS-PAGE followed by immunoblotting. CB indicates cells treated with the p97 inhibitor CB-5083. Relative CYP51A1TM levels are displayed below the anti-HA blot, normalized to the GAPDH loading control.

(B) Lysates of clonal RNF185, MBRL, SPP, UBE2K, and HRD1 KO cells expressing cDNAs encoding either wildtype (WT), a catalytically inactive (CI) mutant, or an empty vector (EV) were subjected to SDS-PAGE followed by immunoblotting. Parental CYP51A1TM-expresing cells, either untreated or treated with the p97 inhibitor CB-5083 (CB), were used as control. Relative CYP51A1TM levels are displayed below the anti-HA blot, normalized to the GAPDH loading control.

(C) ERAD factor dependencies of CYP51A1TM and Erg11TM substrates. Flp-In T-REx 293 cells expressing CYP51A1TM or Erg11TM were transfected with sgRNAs targeting the indicated genes, or a control sgRNA (sgCtrl). Levels of Erg11TM and CYP51A1TM were analyzed by flow cytometry (based on GFP fluorescence).

(D,E) RNF185 and MBRL have unique, non-redundant roles in ERAD. CYP51A1TM levels were analyzed by (D) flow cytometry or immunoblotting (E) in clonal RNF185 and MBRL KO cells expressing cDNAs encoding either wild type RNF185 (RNF185 WT), a catalytically inactive RNF185 mutant (RNF185 CI), MBRL, TEB4 WT, TEB4 CI, or an empty vector (EV). In (E) Tubulin was used as a loading control and detected with an anti-Tubulin antibody.

Supplemental Figure S3

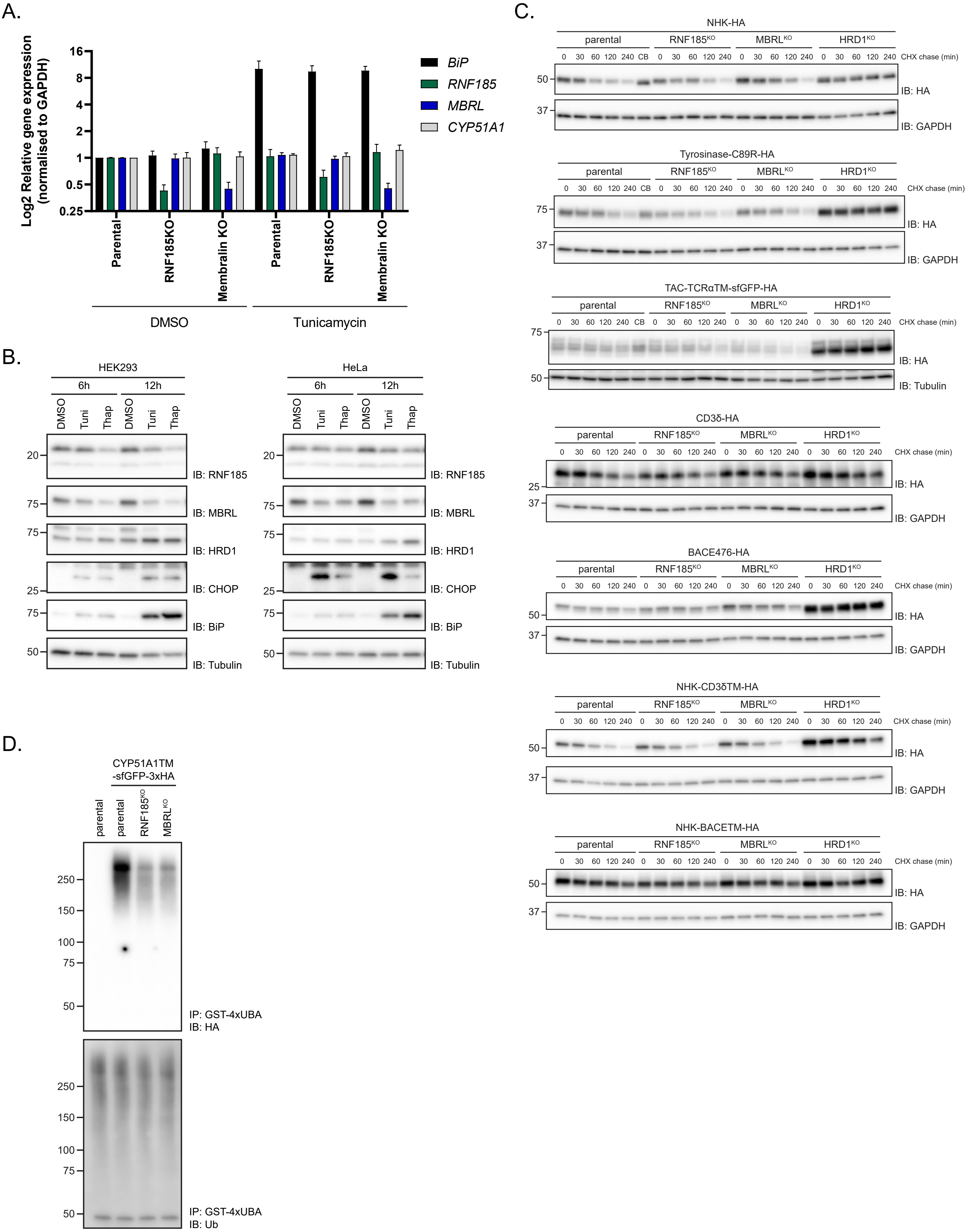

Figure S3: Loss of RNF185 and MBRL does not have a general effect on ER stress and ERAD, related to Figure 3.

(A) RNF185 and MBRL KO cells do not have significant UPR activation and are still capable of mounting an UPR response. T-Rex Flp-In HEK293 parental, RNF185 KO and Membralin KO cells were treated with either DMSO or 2 µg/mL Tunicamycin for 12 hours. Quantitative PCR was performed on *RNF185, TMEM259 (Membralin), CYP51A1* and *GRP78 (BiP)*. The house-keeping gene *GAPDH* was used as a reference. Reactions were performed with three independent biological samples, each analyzed in three technical replicates. Error bars represent the standard deviation.

(B) RNF185 and MBRL protein levels are not increased upon pharmacological UPR activation. HEK293 and HeLa cells were treated with either DMSO, 2 µg/mL Tunicamycin, or 1 mM Thapsigargin for 6 or 12 hours. Cell extracts were analyzed by SDS–PAGE and immunoblotted for the indicated proteins. Tubulin was used as a loading control and detected with an anti-Tubulin antibody.

(C) Degradation of the indicated canonical ERAD substrates was analyzed upon inhibition of protein synthesis with cycloheximide (CHX) in parental, RNF185, MBRL and HRD1 KO cells. Cell extracts were analyzed by SDS–PAGE and immunoblotting.

(D) Ubiquitination of CYP51A1TM is dependent on RNF185 and MBRL. Ubiquitin conjugates from parental, RNF185 KO and MBRL KO cells were isolated using GST-4xUBA. Eluted proteins were analyzed by SDS-PAGE followed by immunoblotting with anti-HA (top) and anti-Ubiquitin (bottom) antibodies. Parental cells not expressing CYP51A1TM were used as control.

Supplemental Figure S4

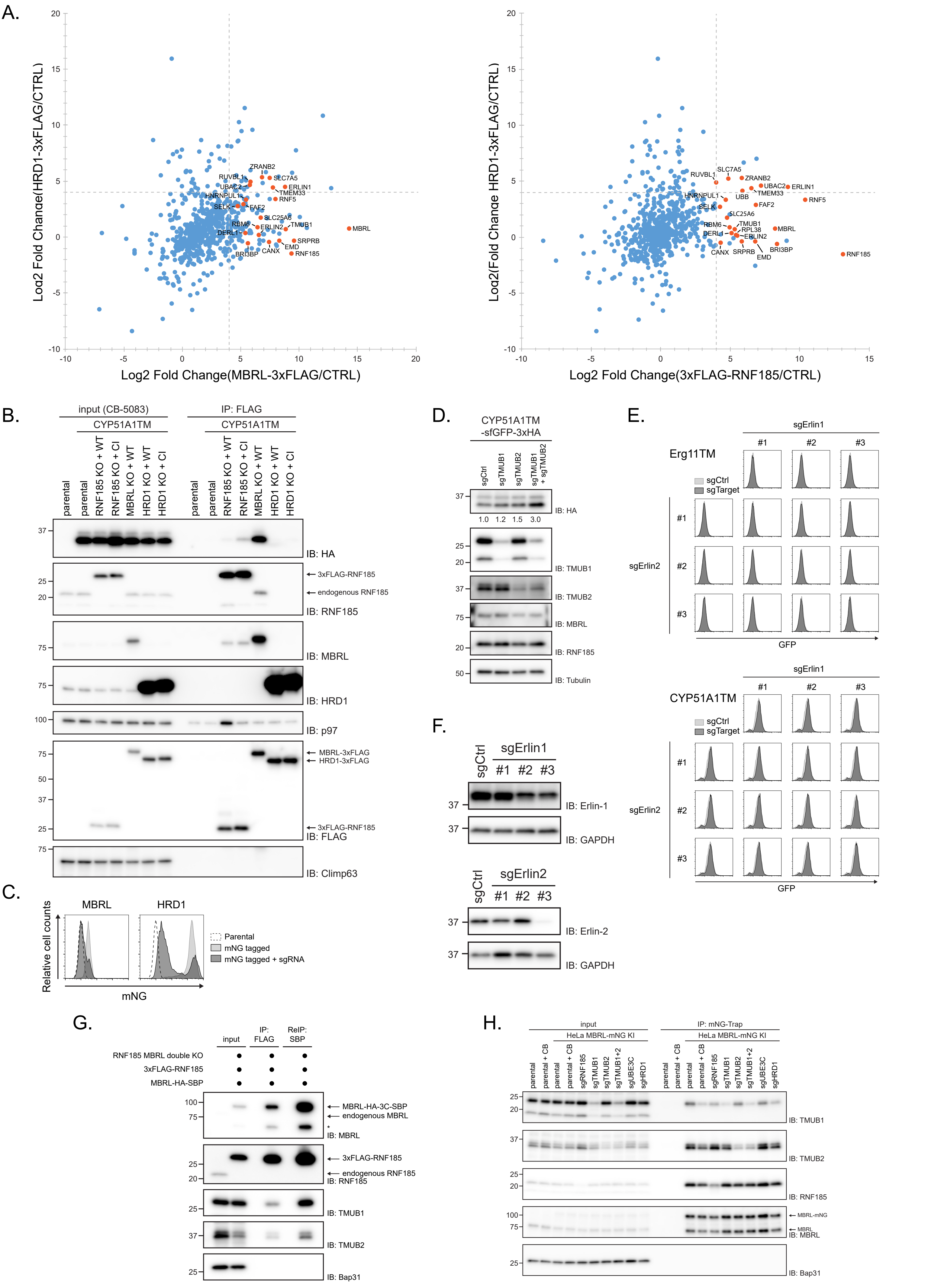

Figure S4: Analysis of the RNF185-Membralin complex, related to Figure 4.

(A) Proteins co-precipitating with MBRL-3xFLAG (x-axis; left graph) or 3xFLAG-RNF185 (x-axis; right graph) versus HRD1-3xFLAG/ctrl (y-axis; both graphs) as analyzed by mass spectrometry. The enrichment of the proteins associated to FLAG-tagged baits over an untagged control was used to calculate the log2 fold changes. The cutoff enrichment was arbitrarily set to 4 (dashed lines). Proteins enriched above the cutoff in both MBRL and RNF185 immunoprecipitates from Figure 4A are annotated and orange colored.

(B) Analysis of proteins co-precipitating with FLAG-tagged RNF185, MBRL and HRD1 by immunoblotting. Parental control cells and the indicated KO cells expressing the corresponding FLAG-tagged proteins treated with the p97 inhibitor CB-5083 (4 h) were lysed and detergent extracts (1% DMNG) subjected to immunoprecipitation with anti-FLAG resin. Eluted proteins were analyzed by SDS-PAGE and immunoblotting. WT, wildtype; CI, catalytically inactive.

(C) Flow cytometric validation of endogenously mNG-tagged MBRL and HRD1. Cells were transfected with sgRNA targeting MBRL or HRD1, after which mNG fluorescence was assessed using flow cytometry. mNeonGreen-negative cells (parental) were included as a baseline control.

(D) Western blot validation of sgRNA-mediated depletion of TMUB1 and TMUB2. Relative CYP51A1TM levels are displayed below the anti-HA blot, normalized to the Tubulin loading control.

(E) Erlin-1 and Erlin-2 are not functionally involved in the degradation of Erg11TM and CYP51A1TM. Cells were depleted for either Erlin-1, Erlin-2, or both using sgRNAs. Levels of CYP51A1TM-sfGFP-3xHA were analyzed by flow cytometry (based on GFP fluorescence).

(F) Western blot validation of sgRNA-mediated depletion of Erlin-1 and Erlin-2.

(G) RNF185, MBRL and TMUB1/2 assemble in a defined biochemical complex, as assessed by sequential immunoprecipitation. Extracts from cells expressing 3xFLAG-RNF185 and MBRL-HA-SBP were immunoprecipitated with anti-FLAG resin followed by native FLAG-peptide elution. Eluates were re-immunoprecipitated with Streptavidin beads. Final elutions were analyzed by SDS-PAGE and immunoblotting with the antibodies directed to the indicated proteins. The asterisk (*) indicates a truncated MBRL product.

(H) RNF185-MBRL complex composition. Endogenous MBRL tagged with mNG on the C-terminus was immunoprecipitated from 1% DMNG lysates of the indicated cell lines. Eluted proteins were analyzed by immunoblotting.

Supplemental Figure S5

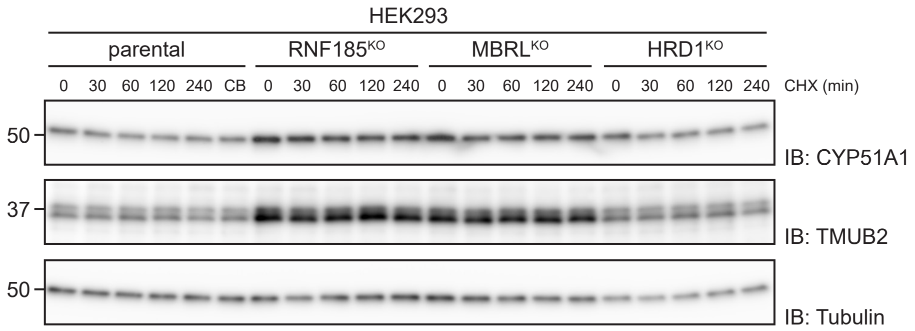

Figure S5: Turnover of CYP51A1 and TMUB2 in RNF185 and MBRL KO cells, related to Figure 5.

The turnover of endogenous CYP51A1 and TMUB2 was assessed by inhibition of protein synthesis using cycloheximide (CHX) for indicated time points in parental, RNF185, MBRL and HRD1 KO cells. Lysates were subjected to SDS-PAGE followed by immunoblotting for the indicated proteins. CB, CB-5083-treated.

Supplemental Figure S6

A.

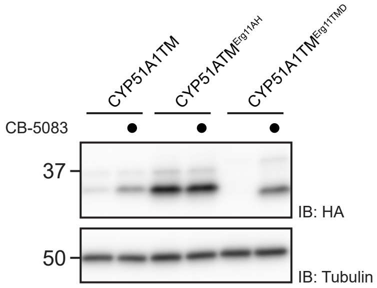

B.

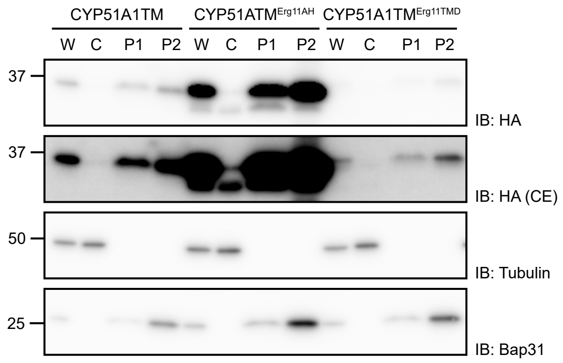

C. CYP51A1TM^{Erg11TMD}-sfGFP-3xHA

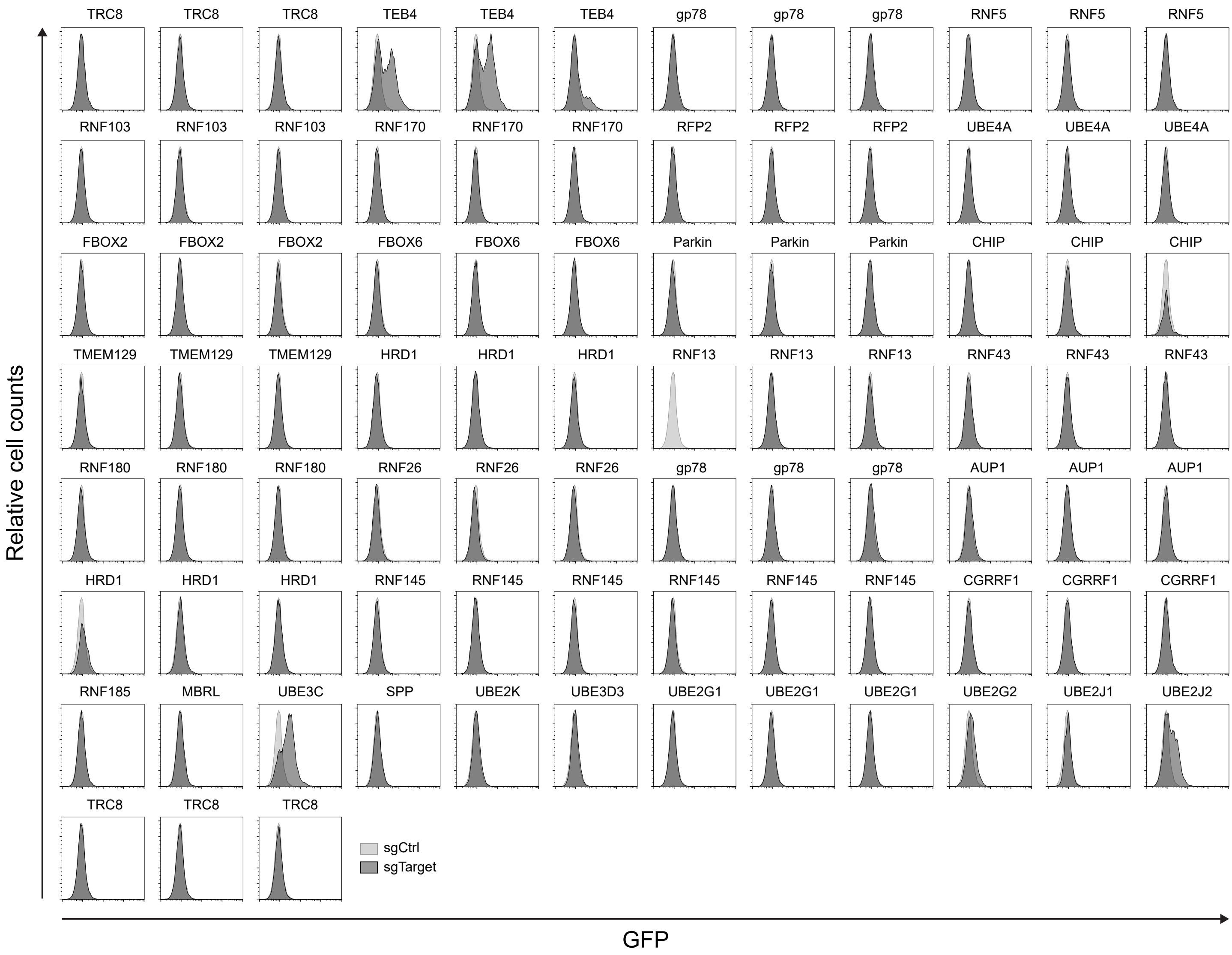

Figure S6: Analysis of CYP51A1TM chimeric constructs, related to Figure 6.

(A) CYP51A1TM and chimeric constructs were expressed in Flp-In T-REx HEK293 cells, after which cells were cultured in the absence or presence of the p97 inhibitor CB-5083 (4 h). Protein levels were assessed by immunoblotting. Tubulin was used as loading control.

(B) CYP51A1TM and its chimeric derivatives are stably associated with membranes, as determined by subcellular fractionation. Postnuclear supernatants (W) were fractionated into a cytosolic soluble fraction (C) and a crude membrane pellet (P1). Membranes in P1 were salt washed and carbonate-treated (P2) to remove all peripherally associated proteins. Fractions were subjected to SDS-PAGE and western blotting analysis. The partitioning of the HA-tagged substrates was compared to endogenous soluble (Tubulin) and integral membrane (BAP31) proteins.

(C) Flp-In T-REx 293 cells expressing CYP51A1TM^{Erg11TMD}-sfGFP-3xHA were transfected with sgRNAs against indicated targets. Levels of CYP51A1TM^{Erg11TMD}-sfGFP-3xHA were analyzed by flow cytometry (based on GFP fluorescence).
