# Supplementary material for: Quality Control of ER Membrane Proteins by the RNF185/Membralin Ubiquitin Ligase Complex
Source: Mol Cell. 2020 Sep 3;79(5):768–781.e7. doi: 10.1016/j.molcel.2020.07.009 (PMC7482433; doi:10.1016/j.molcel.2020.07.009)
Supplement: Document S2. Article plus Supplemental Information [file mmc7.pdf]

Molecular Cell

Article

Quality Control of ER Membrane Proteins by the RNF185/Membralin Ubiquitin Ligase Complex

Graphical Abstract

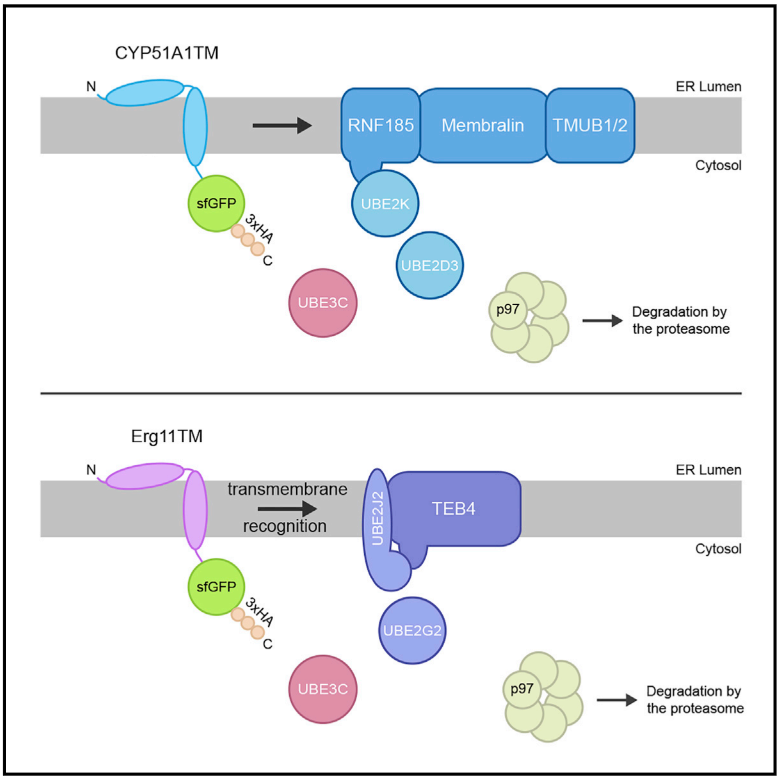

Highlights

- The RNF185 ubiquitin ligase, Membralin, and TMUB1/2 assemble into an ERAD complex
- RNF185/Membralin complex targets membrane proteins, including CYP51A1 and TMUB2
- RNF185/Membralin and TEB4 ERAD complexes recognize distinct substrate features
- TEB4 ERAD complex recognizes substrates through their transmembrane domain

Authors

Michael L. van de Weijer, Logesvaran Krshnan, Sabrina Liberatori, ..., Roman Fischer, Daniel Ebner, Pedro Carvalho

Correspondence

pedro.carvalho@path.ox.ac.uk

In Brief

Membrane proteins are diverse, and the components involved in their quality control in each case are unclear. By comparing the degradation of two short-lived ER proteins, van de Weijer et al. identified an ERAD complex composed of RNF185, TMUBs, and Membralin, a membrane protein essential to neuronal function.

van de Weijer et al., 2020, Molecular Cell *79*, 768–781
September 3, 2020 © 2020 The Author(s). Published by Elsevier Inc.
https://doi.org/10.1016/j.molcel.2020.07.009

CellPress

CellPress
OPEN ACCESS

Molecular Cell

Article

Quality Control of ER Membrane Proteins by the RNF185/Membralin Ubiquitin Ligase Complex

Michael L. van de Weijer,[1] Logesvaran Krshnan,[1] Sabrina Liberatori,[1] Elena Navarro Guerrero,[2] Jacob Robson-Tull,[1] Lilli Hahn,[1] Robert Jan Lebbink,[3] Emmanuel J.H.J. Wiertz,[3] Roman Fischer,[2] Daniel Ebner,[2] and Pedro Carvalho[1,4,*]
[1]Sir William Dunn School of Pathology, University of Oxford, South Parks Road, Oxford OX1 3RE, UK
[2]Nuffield Department of Medicine, Target Discovery Institute, University of Oxford, Oxford OX3 7FZ, UK
[3]Medical Microbiology, University Medical Center Utrecht, 3584 Utrecht, the Netherlands
[4]Lead Contact
*Correspondence: pedro.carvalho@path.ox.ac.uk
https://doi.org/10.1016/j.molcel.2020.07.009

SUMMARY

Misfolded proteins in the endoplasmic reticulum (ER) are degraded by ER-associated degradation (ERAD). Although ERAD components involved in degradation of luminal substrates are well characterized, much less is known about quality control of membrane proteins. Here, we analyzed the degradation pathways of two short-lived ER membrane model proteins in mammalian cells. Using a CRISPR-Cas9 genome-wide library screen, we identified an ERAD branch required for quality control of a subset of membrane proteins. Using biochemical and mass spectrometry approaches, we showed that this ERAD branch is defined by an ER membrane complex consisting of the ubiquitin ligase RNF185, the ubiquitin-like domain containing proteins TMUB1/2 and TMEM259/Membralin, a poorly characterized protein. This complex cooperates with cytosolic ubiquitin ligase UBE3C and p97 ATPase in degrading their membrane substrates. Our data reveal that ERAD branches have remarkable specificity for their membrane substrates, suggesting that multiple, perhaps combinatorial, determinants are involved in substrate selection.

INTRODUCTION

Misfolded proteins in the lumen and membrane of the endoplasmic reticulum (ER) are degraded by ER-associated degradation (ERAD) (Christianson and Ye, 2014; Mehrtash and Hochstrasser, 2019; Wu and Rapoport, 2018). In a signal-dependent manner, ERAD also degrades certain folded proteins, such as rate-limiting enzymes for sterol biosynthesis, thereby controlling the metabolic flux through this vital pathway (Ruggiano et al., 2014). These functions of ERAD in protein and lipid homeostasis are conserved throughout eukaryotes and are relevant to the etiology of several diseases in humans (Guerriero and Brodsky, 2012).

Studies in yeast and mammalian cells showed that, upon recognition, both luminal and membrane substrates are retrotranslocated across the ER bilayer to the cytosol and ubiquitinated (Christianson and Ye, 2014; Ruggiano et al., 2014). These sequential steps are coordinated by ER membrane ubiquitin ligase complexes, each defining an ERAD branch with specificity to certain classes of substrates. The diverse ERAD branches converge on the cytosolic p97 ATPase complex (Cdc48 in yeast), which extracts ubiquitinated substrates from the ER membrane and hands them to the proteasome for degradation (Wu and Rapoport, 2018).

Degradation of ER luminal substrates depends on a single ERAD branch, with all substrates characterized so far being routed to the Hrd1 ubiquitin ligase complex (Carvalho et al., 2006; Christianson et al., 2011; Denic et al., 2006; Gauss et al., 2006). In contrast, all known ERAD branches are able to process membrane substrates. For example, the three ERAD branches in yeast, defined by the Hrd1, Doa10, and Asi complexes, target distinct sets of membrane substrates. Earlier work, using a small number of substrates, found some correlation between the position of the misfolded domain in relation to the ER membrane and the ERAD branch selected (Carvalho et al., 2006; Huyer et al., 2004; Vashist and Ng, 2004). However, it is becoming apparent that such a simple criterium does not explain the selection of a vast and diverse set of membrane substrates (Bernasconi et al., 2010; Foresti et al., 2013; Habeck et al., 2015; Hampton et al., 1996; Natarajan et al., 2020). Further complexity is observed in mammalian cells, where close to ten ubiquitin ligases were shown to be involved in ERAD of membrane proteins. However, as in yeast, it is not clear how substrate selection is determined in each case (Leto et al., 2019; Stefanovic-Barrett et al., 2018). Moreover, there are over a dozen more ER-localized uncharacterized ubiquitin ligases, and whether any of these is involved in ERAD is unknown (Kaneko et al., 2016; Li et al., 2008; Neutzner et al., 2011).

To start dissecting the mechanism of quality control in mammalian cells, we developed and characterized the degradation of two model ERAD membrane substrates, CYP51A1TM

 © 2020 The Author(s). Published by Elsevier Inc.
This is an open access article under the CC BY license (http://creativecommons.org/licenses/by/4.0/).

CellPress
OPEN ACCESS

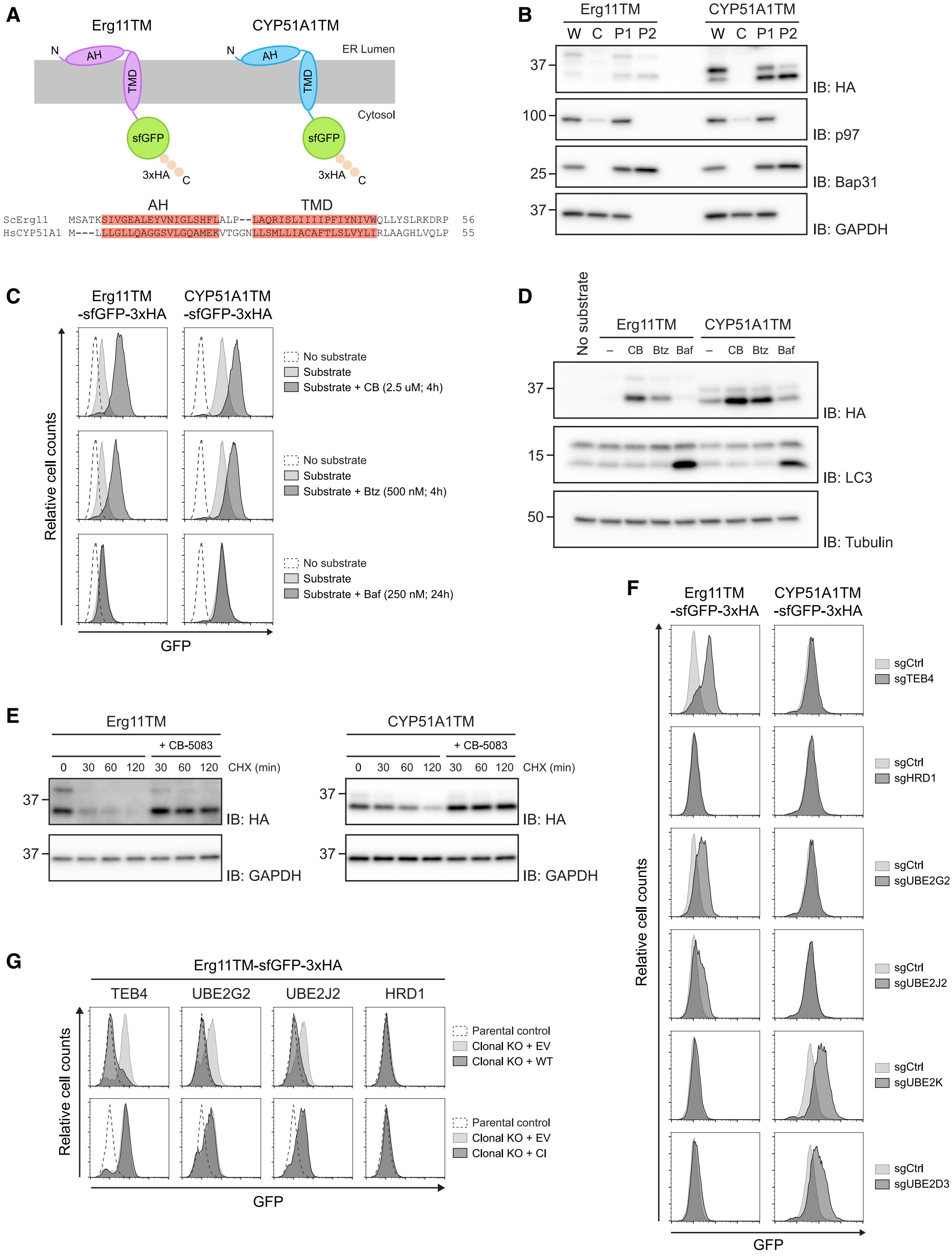

Figure 1. Erg11TM and CYP51A1TM Engage Distinct ERAD Branches

(A) Schematic representation of the Erg11TM and CYP51A1TM constructs. Erg11TM and CYP51A1TM were C-terminally tagged with sfGFP and a 3xHA-tag. The Erg11TM and CYP51A1TM amphipathic helix (AH) and transmembrane (TMD) domain are aligned and highlighted in red.

(B) Subcellular fractionation of Erg11TM- and CYP51A1TM-expressing cells. Postnuclear supernatants (W) were fractionated into a cytosolic soluble fraction (C) and a crude membrane pellet (P1). Membranes in P1 were salt washed and carbonate treated (P2) to remove all peripherally associated proteins. Fractions were subjected to SDS-PAGE and western blotting analysis. The partitioning of the HA-tagged substrates was compared to endogenous soluble (p97 and GAPDH) and integral membrane (BAP31) proteins.

(legend continued on next page)

and Erg11TM. We showed that, despite their seemingly similar domain organization, the two substrates engage distinct ERAD branches. Although Erg11TM was degraded by the TEB4 ubiquitin ligase ERAD complex, abrogation of the best characterized ERAD branches failed to stabilize CYP51A1TM. A forward genetic screen to identify the components involved in CYP51A1TM degradation discovered a previously uncharacterized ERAD branch. This is defined by a complex composed of the ubiquitin ligase RNF185 and the poorly characterized multi-spanning ER membrane proteins TMEM259/Membralin and ubiquitin-like domain (UBL)-containing TMUB1/2. This complex cooperates with UBE3C, a cytosolic ubiquitin ligase recently implicated in ERAD (Leto et al., 2019). Finally, we showed that distinct features are involved in substrate selection by the RNF185/Membralin and TEB4 ERAD branches.

RESULTS

Membrane Domains of Erg11 and CYP51A1 Are ERAD Substrates

In *S. cerevisiae*, the first 68 amino acids of the lanosterol demethylase Erg11, encompassing the membrane domain (hereafter called Erg11TM), are sufficient for its degradation by ERAD (Natarajan et al., 2020). To gain insight on the quality control of membrane proteins in mammalian cells, we tested whether Erg11TM would be an ERAD substrate when expressed in human HEK293 cells. In parallel, we also expressed an analogous construct derived from the human Erg11 homolog CYP51A1 (hereafter called CYP51A1TM; Figure 1A). Like Erg11TM (Monk et al., 2014), CYP51A1TM is predicted to have an N-terminal amphipathic helix (AH) in the ER lumen followed by a single transmembrane alpha helix (TMD). Both Erg11TM and CYP51A1TM were fused to the superfolder green fluorescent protein (sfGFP) and the hemagglutinin (HA) tag for easy detection by flow cytometry, microscopy, and immunoblotting (Figure 1A). Expression of Erg11TM and CYP51A1TM in HEK293 cells showed that they localize to the ER (Figure S1A) and were stably associated with the membrane (Figure 1B). Thus, like their full-length counterparts, Erg11TM and CYP51A1TM are ER integral membrane proteins.

Using the tetracycline-inducible Flp-In T-REx system in HEK293 cells, we analyzed the expression levels of Erg11TM and CYP51A1TM. Upon tetracycline induction, both Erg11TM and CYP51A1TM were detected, and their protein levels were strongly increased upon short treatments with CB-5083 or bortezomib, inhibitors of the p97 ATPase and proteasome, respectively (Figures 1C and 1D). In contrast, treatment with bafilomycin A, which blocks lysosomal degradation, did not affect Erg11TM or CYP51A levels, although it stabilized the lysosomal substrate LC3 (Figures 1C and 1D). Thus, the levels of Erg11TM and CYP51A1TM are controlled by p97 and the proteasome, suggesting that they are ERAD substrates.

To directly analyze Erg11TM and CYP51A1TM turnover, translation shutoff experiments were performed. We observed that both Erg11TM and CYP51A1TM were short-lived proteins with half-lives of approximately 30 and 60 min, respectively (Figure 1E). However, degradation of both proteins was abrogated upon inhibition of p97 with CB-5083, indicating that the ATPase is necessary for Erg11TM and CYP51A1TM extraction from the ER membrane prior to proteasomal degradation. Together, these data indicate that Erg11TM and CYP51A1TM are genuine ERAD substrates.

Erg11TM and CYP51A1TM Follow Distinct ERAD Routes

To identify the ERAD branch(es) involved in Erg11TM and CYP51A1TM degradation, we used a panel of single-guide RNAs (sgRNAs) targeting selected ERAD components, including well-established E3 ubiquitin ligases and E2 ubiquitin-conjugating enzymes (Figures 1F and S1B–S1E). Degradation of Erg11TM was specifically impaired by sgRNAs targeting the ubiquitin ligase TEB4 (Figures 1F, 1G, and S1B) and the ubiquitin-conjugating enzymes UBE2G2 and UBE2J2 (Figures 1F, 1G, and S1D), previously shown to assist TEB4-dependent ubiquitination (Leto et al., 2019; Stefanovic-Barrett et al., 2018). Simultaneous depletion of UBE2G2 and UBE2J2 strongly increased Erg11TM levels to that observed in TEB4-deficient cells, showing that these conjugating enzymes are redundant (Figure S1F).

Curiously, degradation of CYP51A1TM was unaffected upon depletion of canonical ERAD ubiquitin ligases but was impaired by sgRNAs targeting the ubiquitin-conjugating enzymes UBE2K and UBE2D3 (Figures 1F and 1G), whose role in ERAD is not fully understood yet (Chen and Pickart, 1990; Flierman et al., 2006; Leto et al., 2019; van de Weijer et al., 2014). Simultaneous depletion of UBE2K and UBE2D3 strongly stabilized CYP51A1TM, suggesting that these conjugating enzymes are partly redundant (Figure S1G). Thus, although displaying similar domain

(C and D) Erg11TM and CYP51A1TM levels are stabilized upon inhibition of p97 and the proteasome but are unaffected by blocking protein delivery to lysosomes. Flow cytometry (C) and immunoblotting (D) analysis of tetracycline-induced expression of Erg11TM and CYP51A1TM in Flp-In T-REx 293 cells. Analysis was performed 24 h post-induction in cells left untreated, incubated 4 h with inhibitors to p97 (CB-5083; 2.5 μM [CB]) or to the proteasome (Bortezomib; 500 nM [Btz]), or incubated 24 h with bafilomycin A (250 nM [Baf]) that inhibits lysosomal delivery. Flp-In T-REx 293 cells without any GFP-tagged substrate were used as control (no substrate). In (D), Erg11TM and CYP51A1TM were detected with anti-HA antibodies. LC3 was analyzed to confirm effectiveness of bafilomycin A treatment and was detected with an anti-LC3 antibody. Tubulin was used as a loading control and was detected with an anti-tubulin antibody.
(E) Degradation of Erg11TM and CYP51A1TM was analyzed after inhibition of protein synthesis by cycloheximide (CHX) in the absence or presence of the p97 inhibitor CB-5083 (4 h; 2.5 μM). Cell extracts were analyzed by SDS-PAGE and immunoblotting. Erg11TM and CYP51A1TM were detected with anti-HA antibodies. GAPDH was used as a loading control and detected with an anti-GAPDH antibody.
(F) Erg11TM and CYP51A1TM follow distinct ERAD pathways. Cells expressing Erg11TM and CYP51A1TM as well as plasmids encoding sgRNAs targeting the indicated genes were analyzed by flow cytometry (based on GFP fluorescence).
(G) Degradation of Erg11TM depends on TEB4, UBE2G2, and UBE2J2. Erg11TM levels were analyzed by flow cytometry (based on GFP fluorescence) in parental control cells and in clonal TEB4, UBE2G2, UBE2J2, and HRD1 KO cells expressing cDNAs encoding either the corresponding wild type (WT), a catalytically inactive (CI) mutant, or an empty vector (EV).
See also Figure S1.

CellPress
OPEN ACCESS

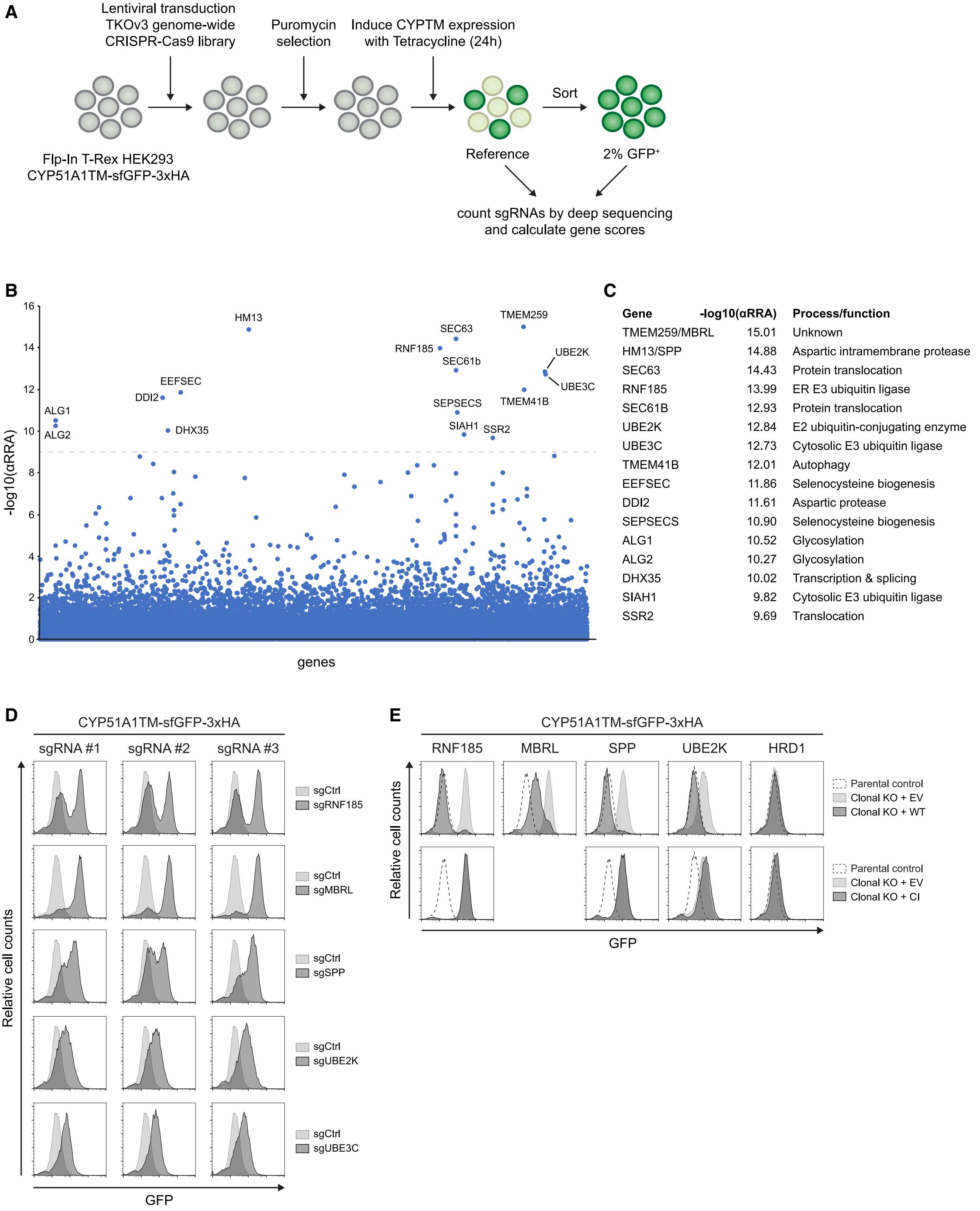

(legend on next page)

organization, CYP51A1TM and Erg11TM appear to follow distinct ERAD routes.

Identification of the Components Involved in CYP51A1TM ERAD

To define the machinery involved in CYP51A1TM ERAD, we performed a genome-wide CRISPR-Cas9 screen. HEK293 Flp-In T-REx cells expressing CYP51A1TM were infected with the Toronto KnockOut CRISPR-Cas9 Library version 3 (TKOv3), which contains a total of 70,948 sgRNAs targeting 18,053 human genes (Hart et al., 2017). The TKOv3 library has an average of 4 sgRNAs per gene, which minimizes the rate of false-negative results and also includes several control sgRNAs (Hart et al., 2017). Mutant cells with high levels of CYP51A1TM were isolated by flow cytometry, and next-generation sequencing was used to sequence and quantify sgRNAs in reference and sorted cell populations (Figure 2A). Gene rankings generated using the MAGeCK algorithm (Li et al., 2014) identified 16 genes that, when perturbed, resulted in a highly significant increase in CYP51A1TM levels (Figures 2B and 2C). Among these was the E2 ubiquitin-conjugating enzyme UBE2K, initially identified in the targeted screen, supporting the specificity of the approach. Several genes encoding for proteins with ER-related functions, such as protein translocation, glycosylation, and autophagosome formation, were also identified, but their involvement in CYP51A1TM degradation is likely indirect and was not further investigated (Figure S2A). The CRISPR screen pinpointed four additional genes, RNF185, UBE3C, signal peptide peptidase (SPP) (also known as HM13), and Membralin (MBRL), which encode proteins with links to ERAD and/or quality control and are characterized below.

To validate the genome-wide CRISPR-Cas9 screen, three independent sgRNAs targeting RNF185, UBE3C, SPP, UBE2K, and MBRL were tested. In all cases, depletion of these proteins resulted in increased CYP51A1TM levels (Figures 2D and S2A). Moreover, we generated HEK293 clonal knockout (KO) cell lines for RNF185, MBRL, SPP, and UBE2K, all of which displayed increased levels of CYP51A1TM, as expected (Figures 2E and S2B). In each case, re-expression of the depleted protein restored CYP51A1TM levels, confirming the specificity of the phenotype. In contrast, re-expression of catalytically inactive RNF185, UBE2K, and SPP had no effect, indicating that enzymatic activity of these proteins is necessary in controlling CYP51A1TM levels (Figures 2E and S2B). Thus, the degradation of CYP51A1TM and Erg11TM involve distinct ER components, as suggested by our earlier data (Figures 1F and S2C).

To directly analyze the effects of RNF185, MBRL, and UBE3C on CYP51A1TM turnover, we performed cycloheximide chase experiments. In control HEK293 Flp-In T-REx cells, CYP51A1TM was a short-lived protein with a half-life of ~60 min. In contrast, CYP51A1TM was a long-lived protein in RNF185 and MBRL KO clonal cell lines (Figure 3A). Consistent with the CRISPR screen data, UBE3C KO cell clones show a milder defect in CYP51A1TM turnover (half-life ~120 min; Figure 3B). Thus, although RNF185 and MBRL are essential for the degradation of CYP51A1TM, UBE3C appears to play a less critical role. Importantly, the effect of RNF185 and MBRL was specific, as deletion of these genes did not affect general ER homeostasis (Figures S3A and S3B) or the degradation of other well-established ERAD substrates (Figure S3C). Our analysis was extended to the E2 ubiquitin-conjugating enzymes UBE2K and UBE2D3. In agreement with our genetic data, we observed a strong delay in CYP51A1TM degradation in UBE2K KO cells although depletion of UBE2D3 had a smaller effect (Figure 3C). Thus, UBE2K is the major E2 contributing to CYP51A1TM degradation.

To further characterize the CYP51A1TM ERAD, we analyzed its ubiquitination state. In control cells, ubiquitinated high-molecular-weight CYP51A1TM species were readily detected (Figures 3D and S3D). Cells lacking the ubiquitin ligase RNF185 showed much reduced ubiquitinated CYP51A1TM, even if the overall levels of the substrate were higher (Figures 3D and S3D). Similar reduction in ubiquitinated CYP51A1TM was observed in KO cells for MBRL, a multi-spanning ER membrane protein of unknown function (Andersson and von Euler, 2002; Chen et al., 2005; Yang et al., 2015). Interestingly, CYP51A1TM was still efficiently ubiquitinated in KO cells for UBE3C, a soluble ubiquitin ligase recently implicated in ERAD (Leto et al., 2019), but the ubiquitin chains appeared shorter (Figure 3D). Assembly of a large portion of these shorter ubiquitin chains still required RNF185 and MBRL, as they were reduced in double-KO cells (Figure 3D). Altogether, our data indicate that RNF185 is the main ubiquitin ligase involved in CYP51A1TM ERAD, a process that also requires MBRL. Although not essential, the soluble ubiquitin ligase UBE3C increases the efficiency of the process by extending ubiquitin chains, perhaps through branching (Leto et al., 2019).

Substrate ubiquitination was also analyzed in cells deficient in the E2 enzymes UBE2K and UBE2D3. In the absence of UBE2K, CYP51A1TM accumulated primarily as a monoubiquitinated species although polyubiquitin conjugates were greatly reduced (Figure 3E). In contrast, depletion of UBE2D3 had at best a negligible effect on CYP51A1TM ubiquitination, suggesting that its contribution to substrate degradation may be indirect. Thus, in agreement with the genetic and turnover data, UBE2K is the most prominent E2 enzyme in CYP51A1TM ERAD. Our data show that UBE2K is critical in building polyubiquitin chains on monoubiquitinated substrate.

Figure 2. A Genome-wide CRISPR-Cas9 Screen Identifies Components Required for CYP51A1TM Degradation

(A) Workflow of the CRISPR-Cas9 genome-wide screen.

(B) Significance score of the genes analyzed in the screen calculated by the MAGeCK algorithm. The x axis represents the genes in alphabetical order. The y axis shows the $-\log(\alpha RRA)$ significance value. The $-\log(\alpha RRA)$ cutoff was arbitrarily set at 9 (dashed line). Significantly enriched genes are annotated.

(C) Overview of the enriched genes identified and their proposed function.

(D) Validation of several screen hits using independent sgRNAs. Levels of CYP51A1TM were analyzed by flow cytometry (based on GFP fluorescence).

(E) Degradation of CYP51A1TM depends on RNF185, MBRL, SPP, and UBE2K. CYP51A1TM levels were assessed by flow cytometry (based on GFP fluorescence) in parental control cells and clonal RNF185, MBRL, SPP, UBE2K, and HRD1 KO cells expressing cDNAs encoding either WT, a CI mutant, or an EV. See also Figure S2.

CellPress
OPEN ACCESS

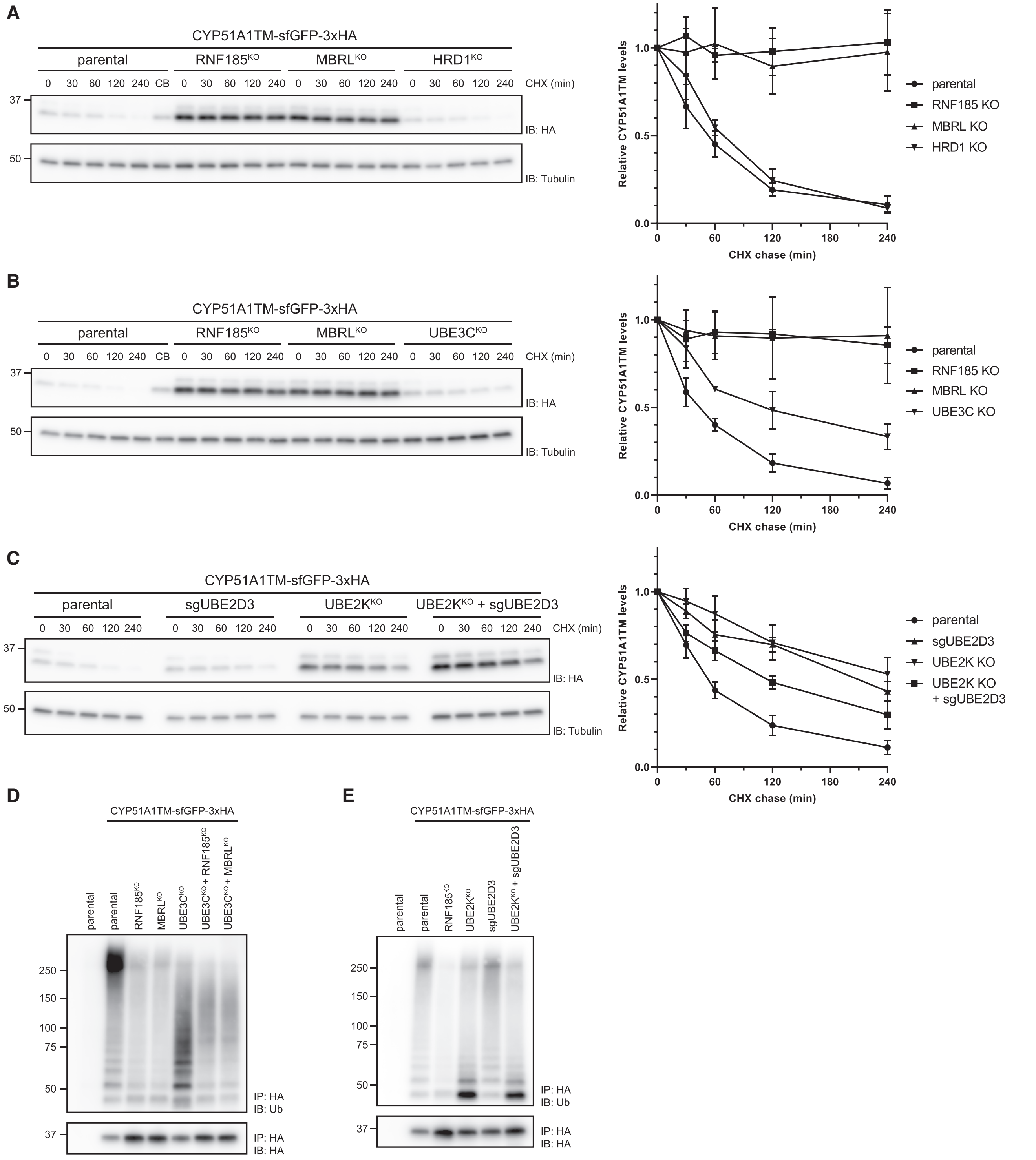

Figure 3. CYP51A1TM Ubiquitination and Degradation Are Dependent on RNF185 and Membralin

(A) RNF185 and Membralin are essential for CYP51A1TM degradation. Degradation of CYP51A1TM was analyzed upon inhibition of protein synthesis with cycloheximide (CHX) in parental, RNF185, MBRL, and HRD1 KO cells. Cell extracts were analyzed by SDS-PAGE and immunoblotting. The graph shows the average of three experiments; error bars represent the standard deviation.

(B) UBE3C promotes efficient CYP51A1TM degradation. Degradation of CYP51A1TM in cells lacking the indicated genes was analyzed as in (A).

(C) The E2s UBE2K and UBE2D3 are involved in CYP51A1TM degradation. Degradation of CYP51A1TM in cells depleted for the indicated genes was analyzed as in (A).

(legend continued on next page)

CYP51A1TM Degradation by the RNF185/MBRL Ubiquitin Ligase Complex

RNF185 and MBRL had similar effects on CYP51A1TM ubiquitination and degradation. We observed that MBRL KO cells had reduced RNF185 protein levels (Figure S2B) although RNF185 transcript levels were unchanged (Figure S3A). However, this is unlikely to explain the defects in CYP51A1TM turnover. The levels of CYP51A1TM in MBRL KO cells were unaffected by restoration of RNF185 levels upon overexpression (Figures S2D and S2E), suggesting that MBRL had a more direct effect on CYP51A1TM ERAD. To explore the relationship between RNF185 and MBRL, we searched for their potential binding partners. HEK293 clonal KO cell lines for RNF185 or MBRL were transduced with lentivirus encoding FLAG-tagged RNF185 and MBRL, respectively. FLAG-tagged proteins were immunoprecipitated from detergent extracts, and the co-precipitated material was analyzed by mass spectrometry. RNF185 and MBRL co-precipitated regardless of which of the two proteins was used as a bait. Moreover, FLAG-RNF185 and MBRL-FLAG shared a similar set of interaction partners (Figures 4A, S4A, and S4B). Importantly, this set of interactors was largely distinct from the one of HRD1, another ERAD ubiquitin ligase (Figure S4A). Taken together, these data suggest that RNF185 and MBRL are part of the same complex.

To ensure that these results were not an artifact of protein overexpression, we analyzed the interaction between endogenous MBRL and RNF185. Using CRISPR-Cas12-mediated genome editing (Fueller et al., 2020), we tagged endogenous MBRL with the mNeonGreen (mNG) fluorescent protein (MBRL-mNG). Western blotting, flow cytometry, and depletion experiments demonstrate that at least one, but not all, MBRL alleles was successfully tagged with mNeonGreen (Figures 4C and S4C). Endogenously tagged MBRL-mNG was immunoprecipitated from cell lysates and interacting proteins analyzed by mass spectrometry (Figure 4B) and western blot (Figure 4C). MBRL-mNG co-precipitated with untagged endogenous MBRL, suggesting that it oligomerizes. Importantly, in comparison with overexpressed MBRL, endogenous MBRL-mNG displayed a more defined set of interacting proteins, consisting of almost exclusively ER membrane proteins (Figure 4B). Again, RNF185 was identified as the main interacting E3 ubiquitin ligase of MBRL. Curiously, the E3 ubiquitin ligase RNF5, which is more than 70% identical to RNF185 (El Khouri et al., 2013), did not precipitate with endogenous MBRL-mNG, highlighting the specificity of the interaction (Figure 4C).

Some ER membrane proteins with previous links to ERAD were present in the precipitates of overexpressed MBRL and RNF185 as well as of endogenous MBRL (Figures 4A and 4B). These included Erlin1, Erlin2, and TMUB1, previously shown to interact with gp78 (Christianson et al., 2011; Jo et al., 2011; Wang et al., 2009), as well as TMUB2, a poorly characterized TMUB1 paralog. These mass spectrometry results were confirmed in parallel immunoprecipitation experiments followed by immunoblotting (Figure 4C). Close inspection of the genome-wide screen dataset showed that TMUB1 and TMUB2 were identified as weak hits (ranked 32th and 35th, respectively). This suggested that TMUB1 and TMUB2 either had a small contribution in CYP51A1TM degradation or that there was redundancy between these homologous proteins. To distinguish between these possibilities, we re-evaluated the role of TMUB1/2 in CYP51A1TM degradation upon their individual or simultaneous depletion. TMUB1 or TMUB2 single deletion resulted in only marginal increase of CYP51A1TM levels, in agreement with our genetic screen. In contrast, double TMUB1/2 KO cells displayed considerably higher levels of CYP51A1TM (Figures 4D and S4D), indicating that TMUB1/2 have redundant but essential roles in CYP51A1TM degradation. Similar experiments showed that simultaneous depletion of Erlin1/2 did not affect CYP51A1TM levels, suggesting that their interaction with RNF185 and MBRL is not essential for the ERAD of at least some substrates (Figures S4E and S4F).

Sequential immunoprecipitation experiments in RNF185/MBRL double-KO cells virally co-transduced with FLAG-RNF185 and MBRL tagged with streptavidin binding peptide (MBRL-SBP) supported that these proteins form a complex that also contains TMUB1/2 (Figure S4G). Importantly, a similar complex was observed with the endogenous proteins. Size exclusion chromatography of detergent extracts from cells expressing endogenously tagged MBRL-mNG showed that MBRL and RNF185 eluted in a single peak (Figure 4E). This peak was distinct from the ones containing the previously characterized HRD1 and gp78 complexes (Christianson et al., 2011; Hwang et al., 2017) and contained a substantial fraction of TMUB1 and TMUB2. Immunoprecipitation of MBRL-mNG from the size exclusion peak fraction (fraction 9) resulted in the co-precipitation of RNF185, TMUB1, and TMUB2 (Figure 4F). Importantly, other proteins present in small amounts in fraction 9, such as RNF5 and HRD1, did not co-precipitate with MBRL. Thus, endogenous RNF185, MBRL, and TMUBs assemble into a defined biochemical complex, which we called the RNF185/MBRL complex. To get insight into the organization of this complex, we analyzed how its composition was affected by deletion of individual subunits. The interaction of MBRL with RNF185 and TMUBs was unaffected in cells lacking TMUBs and RNF185, respectively (Figure S4H). In contrast, the interaction between RNF185 and TMUBs was strongly reduced in MBRL KO cells (Figure 4G). These data suggest that MBRL bridges the interaction between RNF185 and TMUBs and is the core component of the complex.

Levels of Endogenous CYP51A1 and TMUB2 Are Controlled by the RNF185/MBRL Complex

Although the RNF185/MBRL complex is essential for the degradation of CYP51A1TM model substrate, we wondered whether

(D) Ubiquitination of CYP51A1TM is dependent on RNF185 and MBRL. CYP51A1TM was immunoprecipitated from parental cells or cells lacking the indicated genes and analyzed by SDS-PAGE followed by immunoblotting with anti-HA and anti-ubiquitin antibodies. Parental cells lacking CYP51A1TM substrate were used as a negative control.

(E) UBE2K facilitates polyubiquitination of CYP51A1TM. CYP51A1TM ubiquitination in cells depleted for the indicated E2 enzymes was analyzed as in (D).
See also Figure S3.

CellPress
OPEN ACCESS

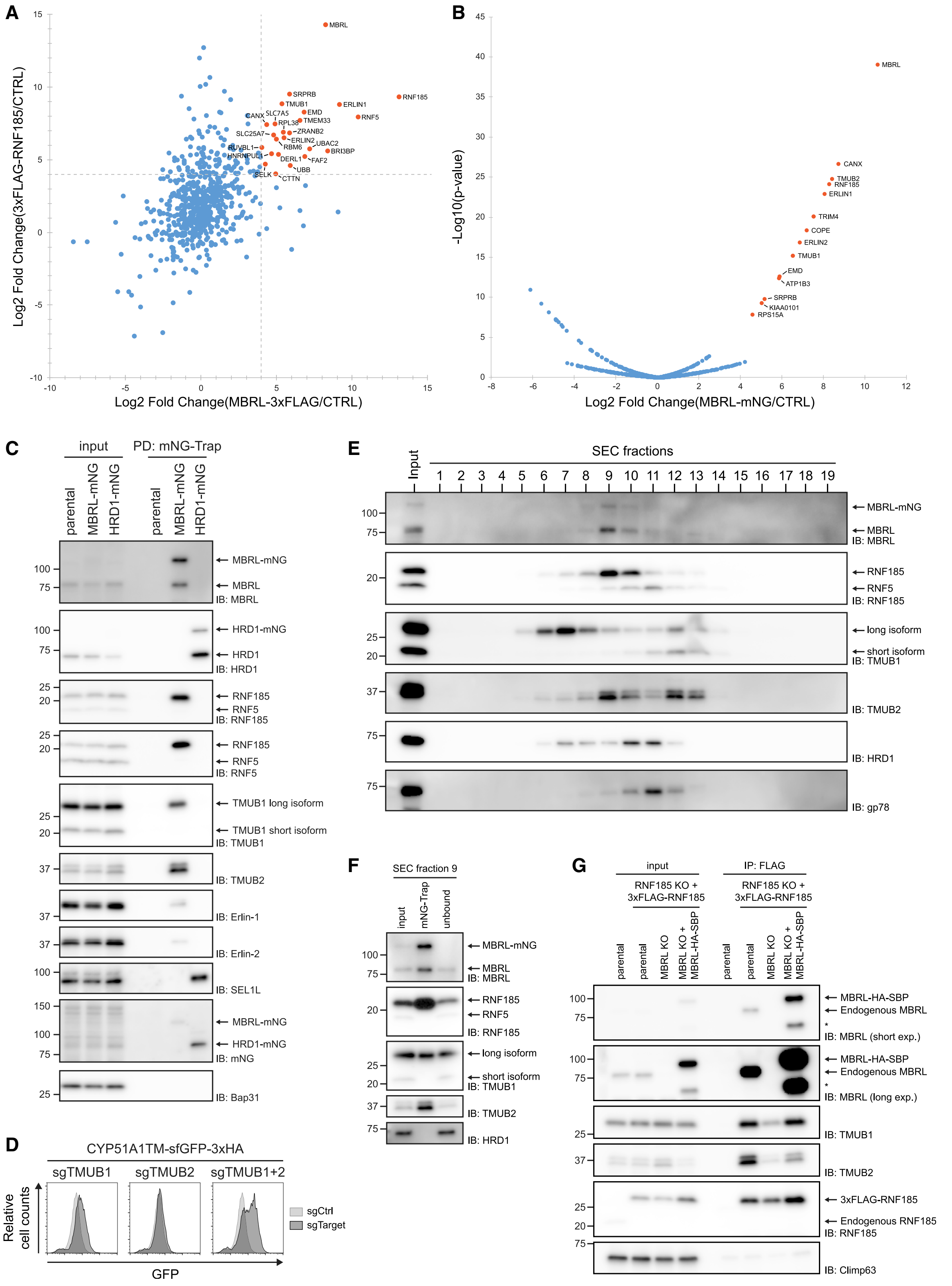

(legend on next page)

this ERAD branch could also degrade endogenous proteins. To start addressing this issue, we analyzed whether the levels of endogenous, full-length CYP51A1 were affected in cells lacking RNF185 and MBRL. Although endogenous CYP51A1 is a long-lived protein (Figure S5), its levels were increased in both HEK293 and HeLa cells lacking RNF185 or MBRL (Figures 5A and 5B). CYP51A1 transcript levels were comparable in control and KO cells, suggesting that the stabilization resulted from defective protein degradation (Figure S3A). Consistent with this possibility, re-expression of RNF185 largely restored normal CYP51A1 levels, although a catalytically inactive RNF185 RING finger mutant had no effect (Figures 5A and 5B). Next, we analyzed whether endogenous CYP51A1 interacted with components of the RNF185/MBRL complex. In general, interactions between ERAD complexes and their substrates are difficult to capture. A commonly used strategy to capture these transient interactions is by curbing substrate processing, for example, with p97 inhibitors (Huang et al., 2018). Alternatively, mutations that render E3 ligases catalytically inactive allow them to bind more stably to substrates, often functioning as substrate "traps." Indeed, we observe that both wild-type RNF185 in the presence of p97 inhibitors and, in particular, a catalytically inactive RNF185 associate with endogenous CYP51A1, as detected by mass spectrometry (Figure 5C). A substantial enrichment of CYP51A1 was also detected in MBRL precipitates (Figure 5C). Interestingly, a similar pattern was observed for the interaction of RNF185 and MBRL with their bona fide substrate CYP51A1TM (Figure S4B). In both cases, we could not detect association with the ERAD ubiquitin ligase HRD1, indicating that the interactions were specific (Figures 5C and S4B). Altogether, these data strongly suggest that endogenous CYP51A1 is a substrate of the RNF185/MBRL complex.

We found that the levels of TMUB2, an integral member of the complex, were also controlled by RNF185 and MBRL. As in the case of CYP51A1, regulation of TMUB2 levels also depended on catalytic activity of RNF185, consistent with the possibility of it being a direct substrate of this ubiquitin ligase (Figures 5A and 5B). Interestingly, the TMUB2 homolog TMUB1 was mostly refractory to RNF185/MBRL regulation (Figures 5A and 5B). There is well-established precedent for subunits of ERAD ubiquitin ligase complexes being substrates of the same complex (Carvalho et al., 2006; Claessen et al., 2010; Swanson et al., 2001; Walter et al., 2001), perhaps as part of a feedback regulation mechanism.

RNF185/MBRL and TEB4 ERAD Complexes Recognize Distinct Features on Their Membrane Substrates

Finally, we exploited the similarities in domain organization between CYP51A1TM and Erg11TM to investigate the determinants involved in substrate recognition by the RNF185/MBRL complex. To this end, we generated chimeric proteins in which the main structural elements within CYP51A1TM, the AH and TMD, were swapped with the corresponding elements from Erg11TM (Figure 6A). The chimeras were integrated in HEK293 cells using the Flp-In T-REx system, and their stability was assessed by flow cytometry in the absence and presence of the p97 inhibitor CB-5083. The CYP51A1TM chimera containing Erg11 AH (CYP51A1TM^{Erg11AH}) was a stable protein and no more a substrate for ERAD. In contrast, the CYP51A1TM chimera containing Erg11 TMD (CYP51A1TM^{Erg11TMD}) was still unstable and stabilized in presence of the p97 inhibitor (Figures 6B and S6A).

Like CYP51A1TM, the chimera CYP51A1TM^{Erg11TMD} was targeted to the ER, and it stably associated with membranes, as assayed through fractionation experiments (Figure S6B). Therefore, to further characterize the degradation of the CYP51A1TM^{Erg11TMD} chimera, we analyzed its turnover in cells deficient for several ERAD branches (Figure S6C). Surprisingly, the levels of CYP51A1TM^{Erg11TMD} were not affected in RNF185 KO cells (Figure 6C). Depletion of MBRL or UBE2K also did not stabilize CYP51A1TM^{Erg11TMD}, indicating that the presence of Erg11TMD was sufficient to switch ERAD branch (Figure S6C). Indeed, the degradation of CYP51A1TM^{Erg11TMD} chimera was reduced in cells lacking either TEB4 or UBE3C (Figures 6C and 6D). Simultaneous ablation of TEB4 and UBE3C resulted in a further increase of CYP51A1TM^{Erg11TMD} steady-state levels (Figure 6C), even if the turnover rate in the double-KO cells was not further reduced (Figure 6D). Together, these data indicate that TEB4 primarily recognizes its substrates through their transmembrane segment. In contrast, multiple features in the membrane and ER lumen appear important for substrate recognition by RNF185/MBRL.

Figure 4. RNF185 and Membralin Form a Novel ERAD Complex

(A) Proteins co-precipitating with MBRL-3xFLAG (x axis) and 3xFLAG-RNF185 (y axis) as analyzed by mass spectrometry. The enrichment of proteins associated to FLAG-tagged baits over an untagged control was used to calculate the log2 fold changes. Proteins enriched above an arbitrary cutoff of 4 in both MBRL and RNF185 IPs are annotated (in orange).

(B) Proteins co-precipitating with endogenous Membralin-mNG as detected by mass spectrometry. The x axis shows the log2 fold change of MBRL-mNG versus untagged control cell line; the y axis shows the −log10 p value estimated by the SignificanceB analysis (Cox and Mann, 2008). Cutoff was arbitrarily set to a log2 (fold change) of 4 with a −log10 (p) of 5. Significantly enriched proteins are annotated (in orange).

(C) Western blotting validation of the interactions identified in (B) by mNG-Trap pull-down (PD) of endogenously tagged Membralin-mNG. Endogenously tagged HRD1-mNG was used as specificity control.

(D) Analysis of CYP51A1TM levels in cells depleted for TMUB1, TMUB2, or both. CYP51A1TM was analyzed by flow cytometry (based on GFP fluorescence).

(E) Size exclusion chromatography of detergent-solubilized membranes of HeLa cells containing endogenously tagged MBRL-mNG (MBRL-mNG). Crude membranes were solubilized in 1% DDM + 0.1% CHS, and solubilized material was applied to a Superose 6 10/300 GL column. Elution fractions were analyzed by SDS-PAGE and western blotting with the indicated antibodies.

(F) Fraction 9 from (E) was subjected to immunoprecipitation using mNG-Trap beads. Eluted material was analyzed by western blotting for the proteins indicated.

(G) Membralin mediates the interaction between RNF185 and TMUBs. Immunoprecipitation of 3xFLAG-RNF185 in cells with the indicated gene deletions is shown. Eluted proteins were analyzed by SDS-PAGE and immunoblotting for the proteins indicated. The asterisk (*) indicates a truncated MBRL product.

See also Figure S4.

CellPress
OPEN ACCESS

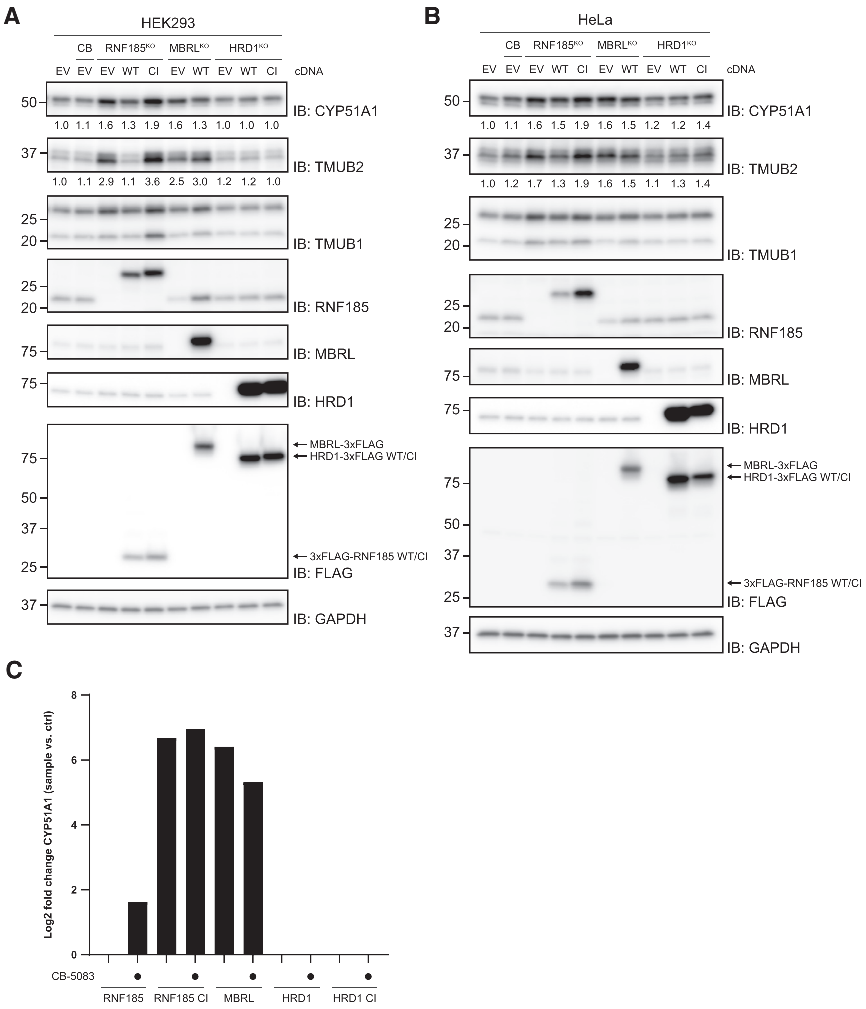

Figure 5. Levels of Endogenous CYP51A1 and TMUB2 Are Regulated by the RNF185-MBRL Complex

(A and B) Protein extracts from RNF185, MBRL, and HRD1 KO (A) HEK293 and (B) HeLa cells expressing either an EV or cDNAs encoding the WT or CI version of the indicated protein were analyzed by SDS-PAGE and immunoblotting. Extracts from parental cells left untreated or treated with the p97 inhibitor CB-5083 (CB) (4 h; 2.5 μM) were also analyzed. Relative CYP51A1 and TMUB2 levels are displayed below the respective blot, normalized to the GAPDH loading control.

(C) Endogenous CYP51A1 co-precipitates specifically with RNF185/MBRL complex. The indicated FLAG-tagged proteins (x axis) were precipitated from untreated or CB-5083-treated cells, and eluted proteins were analyzed by mass spectrometry. The y axis shows the log2 fold enrichment of endogenous CYP51A1 in the FLAG precipitates versus untagged control.

See also Figure S5.

DISCUSSION

Using unbiased genetic and proteomic approaches, we discovered a new ERAD branch involved in the quality control of a subset of ER integral membrane proteins. This branch is defined by the RING-type ubiquitin ligase RNF185, the UBL-containing proteins TMUB1/2, and MBRL, a conserved protein of unknown function. These proteins assemble into a complex that we named the RNF185/MBRL complex (Figure 7).

We showed that this complex promotes the degradation of the model substrate CYP51A1TM as well as the endogenous ER membrane proteins CYP51A1 and TMUB2. Previous proteomics studies identified RNF185 as a partner of the universal ERAD factor p97 (Hülsmann et al., 2018), which we also observed (Figure S4B). This interaction is particularly prominent in a p97 substrate-trapping mutant, suggesting that RNF185-p97 association is transient and regulated by substrates (Hülsmann et al., 2018). Thus, although the complete range of substrates processed by the RNF185/MBRL complex is currently unknown, it will likely be much larger.

Scattered observations previously linked the individual components of the RNF185/MBRL complex to ERAD. RNF185 was implicated in the degradation of the membrane proteins ARL6IP5 (also known as JWA; Qiu et al., 2018) and the cystic fibrosis transmembrane conductance regulator (CFTR) (El Khouri et al., 2013). Although RNF5 appears to be the major ubiquitin ligase in CFTR quality control (Younger et al., 2006), CFTR folding is inefficient and several ubiquitin ligases were implicated in its quality control (Morito et al., 2008; Ramachandran et al., 2016), including RNF185. RNF185-mediated CFTR turnover appears to involve the conjugating enzymes UBE2J1 and UBCH5 (El Khouri et al., 2013). Although our analysis identified UBE2K and UBE2D3 as the conjugating enzymes assisting RNF185/MBRL-mediated ubiquitination, it is possible that RNF185 engages distinct conjugating enzymes to process its various substrates, a possibility that should be directly tested in the future. It will also be interesting to assess whether RNF185-mediated degradation of CFTR and ARL6IP5 requires its binding partners MBRL and TMUBs.

TMUBs contain two (or three) transmembrane domains proximal to the N and C termini separated by an extended cytosolic region containing a UBL domain of unknown function (Jo et al., 2011; Yang et al., 2008). Proteomics studies identified TMUB1 as a binding partner of gp78 (Christianson et al., 2011; Jo et al., 2011), a well-characterized ERAD ubiquitin ligase (Fang et al., 2001). Consistently, TMUB1 depletion inhibited the sterol-regulated degradation of high-mobility group (HMG)-coenzyme A (CoA) reductase, a gp78 substrate. Whether other gp78 substrates also require TMUB1 is unknown. These observations, together with our results, suggest that TMUB1, and perhaps TMUB2, are part of various ERAD complexes.

Among the RNF185/MBRL complex components, MBRL is the one with the highest number of transmembrane segments (6–8 predicted). Although its function is unknown, ablation of MBRL

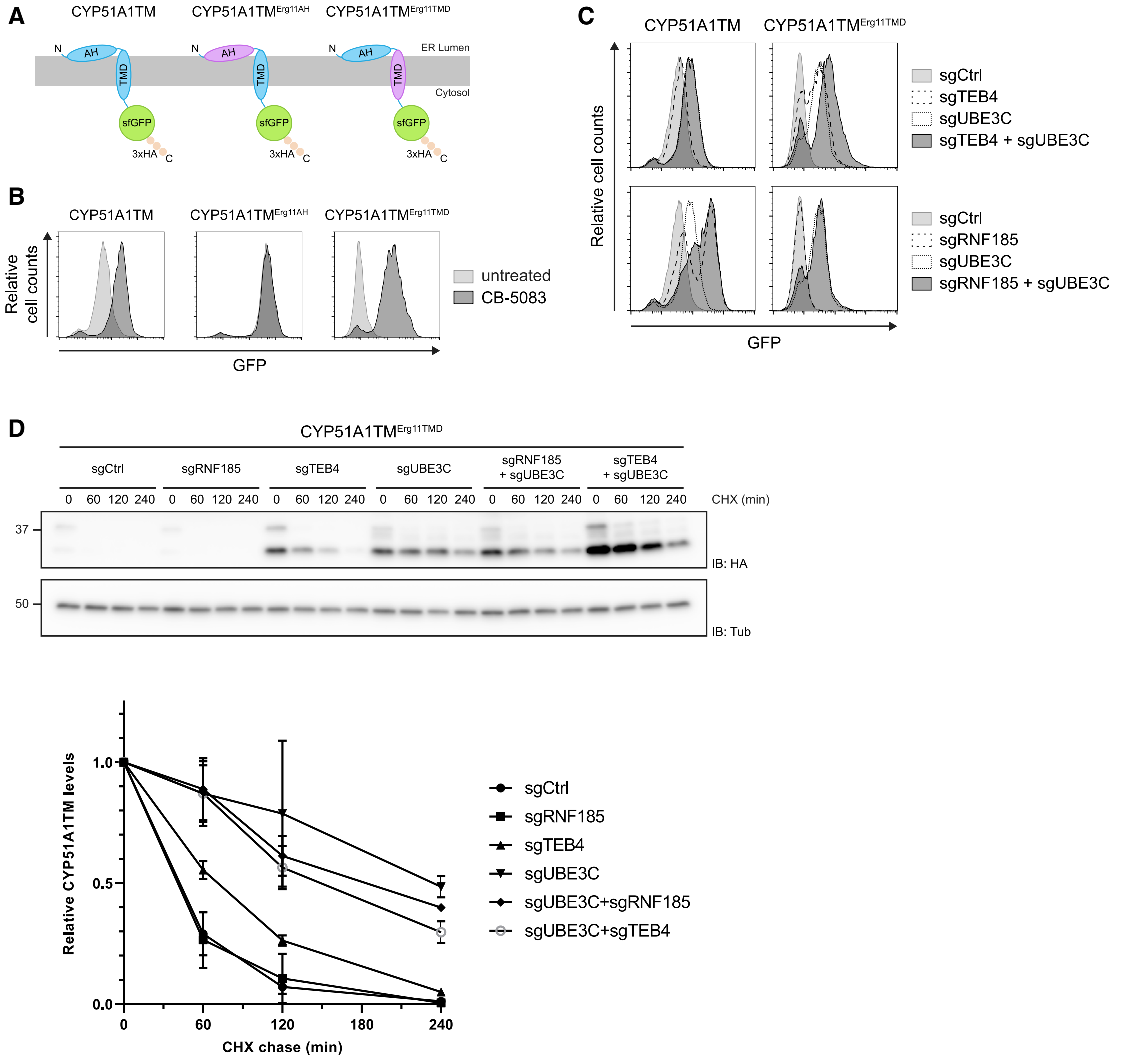

Figure 6. RNF185-MBRL and TEB4 Recognize Distinct Features on Their Membrane Substrates

(A) Schematic overview of the CYP51A1TM chimeras. The AH and the TMD from the Erg11TM ERAD substrate are indicated in purple.

(B) The levels of CYP51A1TM chimeras were analyzed by flow cytometry (based on GFP fluorescence) in the absence or presence of the p97 inhibitor CB-5083 (4 h; 2.5 μM).

(C) The levels of CYP51A1TM and the chimera CYP51A1TM^{Erg11TMD} were analyzed by flow cytometry (based on GFP fluorescence) in cells depleted for the indicated genes.

(D) Degradation of CYP51A1TM^{Erg11TMD} analyzed upon inhibition of protein synthesis with CHX in cells depleted for the indicated genes. Cell extracts were analyzed by SDS-PAGE and immunoblotting. The graph shows the average of three experiments; error bars represent the standard deviation.

See also Figure S6.

in mice is perinatally lethal (Jiang et al., 2019; Yang et al., 2015). Curiously, re-expression of MBRL specifically in astrocytes is sufficient to rescue the lethality in the KO animals, highlighting the essential function of MBRL in the central nervous system. Amyotrophic lateral sclerosis mouse models and patient spinal cord samples showed decreased MBRL expression, suggesting that its function may be related to the etiology of this neurodegenerative disease (Jiang et al., 2019). Understanding how loss of MBRL in the ER gives rise to these phenotypes is of paramount importance. It will also be interesting to investigate how MBRL-binding partners described here contribute to its key functions in the brain.

Studies in cultured cells suggested that MBRL associates with the HRD1 and gp78 ERAD complexes to facilitate the degradation of various proteins, such as NHK, CD3δ, and nicastrin (Zhu et al., 2017). However, we were unable to reproduce those observations. The origin of the discrepancies is unclear, but the convergence of our unbiased genetic and proteomic approaches together with the biochemical experiments in two cell lines unambiguously show that the main functional partners of MBRL are RNF185 and the TMUBs. Our data further suggest that MBRL scaffolds the interaction between RNF185 and TMUBs. Moreover, it interacts robustly with its substrates and, when overexpressed,

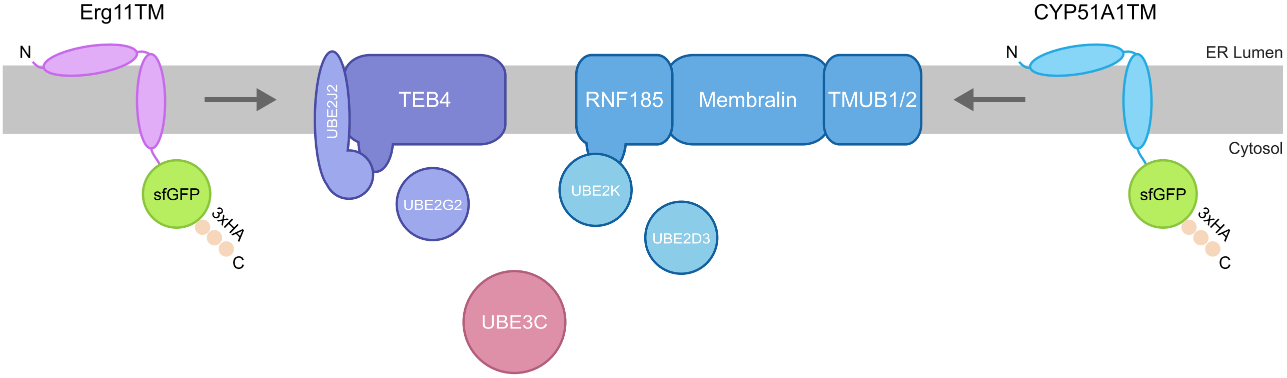

Figure 7. Erg11TM and CYP51A1TM Degradation by the TEB4 and RNF185-MBRL Ubiquitin Ligase Complex
Schematic overview of Erg11TM and CYP51A1TM degradation by the TEB4 and RNF185-MBRL ubiquitin ligase complex, respectively.

inhibits their degradation, possibly by titrating them away from functional complexes. These observations suggest that MBRL plays a role in membrane substrate recognition.

Efficient degradation of the model substrate CYP51A1TM by the RNF185/MBRL complex also requires UBE3C, a cytosolic HECT ubiquitin ligase. Similarly, efficient degradation of the Erg11TM, mainly a TEB4 substrate, also requires UBE3C (Figure 7). These findings are consistent with a recent report implicating UBE3C in the ERAD of some, but not all, substrates (Leto et al., 2019). It was proposed that UBE3C's ability to assemble K29-linked chains (Michel et al., 2015) on substrates previously modified with K48-linked ubiquitin results in heterotypic chains, which may be preferentially processed by the proteasome (Yau et al., 2017). Curiously, our genetic data suggest that UBE3C may have distinct cooperation modes with the RNF185/MBRL and TEB4 ERAD branches. Although TEB4 and UBE3C appear to have independent and additive effects on substrate degradation, this is not the case for the RNF185/MBRL branch. Here, UBE3C appears to work downstream of the RNF185/MBRL complex. However, we were unable to detect any biochemical interaction between the two ubiquitin ligases.

Our analysis of two simple, topologically similar model substrates reveals the complex nature of substrate recognition during membrane protein quality control. The results from the chimeric substrates show that recognition by TEB4 involves the substrate's transmembrane segment. This is in line with recent data implicating TEB4 in ERAD of tail-anchored membrane proteins, a process shared with another ERAD branch mediated by the TRC8 ligase (Stefanovic-Barrett et al., 2018). Curiously, we found no role for TRC8 in Erg11TM degradation, suggesting that the redundancy with TEB4 is limited to some substrates (Figure S1B). Perhaps specific features in the transmembrane segment of Erg11TM, such as the helical destabilizing proline, are preferentially recognized by TEB4. In the case of the RNF185/MBRL complex, neither the transmembrane nor the luminal amphipathic helix of CYP51A1TM was, in isolation, sufficient for substrate recognition, suggesting that it involves multiple determinants. Such a mechanism has been shown to occur during recognition of luminal ERAD substrates, and it was proposed that monitoring of multiple determinants increases the fidelity of protein quality control (Denic et al., 2006; Xie et al., 2009). However, a more detailed analysis including a larger number of substrates will be required to clarify the mechanism of membrane protein recognition during ERAD.

STAR★METHODS

Detailed methods are provided in the online version of this paper and include the following:

- KEY RESOURCES TABLE
- RESOURCE AVAILABILITY
  - Lead Contact
  - Materials Availability
  - Data and Code Availability
- EXPERIMENTAL MODEL AND SUBJECT DETAILS
- METHOD DETAILS
  - Lentivirus production
  - Plasmids
  - Genome-wide CRISPR/Cas9 screen
  - Generation of CRISPR/Cas9-mediated knockout cells
  - Endogenous tagging
  - Co-immunoprecipitations
  - Substrate ubiquitination experiments
  - Translation shut-off experiments
  - Immunoblotting
  - Size-exclusion
  - Mass Spectrometry
  - Fluorescence microscopy
  - Membrane isolation
  - Quantitative PCR analysis
  - Flow cytometry
- QUANTIFICATION AND STATISTICAL ANALYSIS

SUPPLEMENTAL INFORMATION

Supplemental Information can be found online at https://doi.org/10.1016/j.molcel.2020.07.009.

ACKNOWLEDGMENTS

We thank J. Christianson for reagents and discussions, M. Gyrd-Hansen for the plasmid encoding GST-4xUBA from Ubiquilin, D. Simms for advice on

bioinformatics analysis, L. Witty for help with high-throughput sequencing, M. Maj and L. Eriksen for help with flow cytometry, and X. Saayman and A. Vaughan-Jackson for help with qPCR. P.C. was supported by an investigator award from The Wellcome Trust (202642/Z/16/Z).

AUTHOR CONTRIBUTIONS

M.L.v.d.W. and P.C. designed the study. M.L.v.d.W. performed most of the experiments with the help of L.K., J.R.-T., and L.H.; S.L. performed the mass spectrometry analysis with the help of R.F.; E.N.G. and D.E. helped with the optimization of the genome-wide screening; and M.L.v.d.W., R.J.L., and E.J.H.J.W. generated the tailored sgRNA library. M.L.v.d.W. and P.C. analyzed the data with the help of all the authors. P.C. wrote the manuscript with input from all the authors.

DECLARATION OF INTERESTS

The authors declare no competing interests.

Received: December 16, 2019
Revised: May 6, 2020
Accepted: July 7, 2020
Published: July 31, 2020

REFERENCES

Andersson, O., and von Euler, G. (2002). Characterization and expression of the gene encoding membralin, an evolutionary conserved protein expressed in the central nervous system. Brain Res. Gene Expr. Patterns *1*, 205–212.

Bernasconi, R., Galli, C., Calanca, V., Nakajima, T., and Molinari, M. (2010). Stringent requirement for HRD1, SEL1L, and OS-9/XTP3-B for disposal of ERAD-LS substrates. J. Cell Biol. *188*, 223–235.

Carvalho, P., Goder, V., and Rapoport, T.A. (2006). Distinct ubiquitin-ligase complexes define convergent pathways for the degradation of ER proteins. Cell *126*, 361–373.

Chen, Z., and Pickart, C.M. (1990). A 25-kilodalton ubiquitin carrier protein (E2) catalyzes multi-ubiquitin chain synthesis via lysine 48 of ubiquitin. J. Biol. Chem. *265*, 21835–21842.

Chen, Y.-C., Davidson, B., Cheng, C.-C., Maitra, A., Giuntoli, R.L., 2nd, Hruban, R.H., Wang, T.-L., and Shih, IeM. (2005). Identification and characterization of membralin, a novel tumor-associated gene, in ovarian carcinoma. Biochim. Biophys. Acta *1730*, 96–102.

Christianson, J.C., and Ye, Y. (2014). Cleaning up in the endoplasmic reticulum: ubiquitin in charge. Nat. Struct. Mol. Biol. *21*, 325–335.

Christianson, J.C., Olzmann, J.A., Shaler, T.A., Sowa, M.E., Bennett, E.J., Richter, C.M., Tyler, R.E., Greenblatt, E.J., Harper, J.W., and Kopito, R.R. (2011). Defining human ERAD networks through an integrative mapping strategy. Nat. Cell Biol. *14*, 93–105.

Claessen, J.H.L., Mueller, B., Spooner, E., Pivorunas, V.L., and Ploegh, H.L. (2010). The transmembrane segment of a tail-anchored protein determines its degradative fate through dislocation from the endoplasmic reticulum. J. Biol. Chem. *285*, 20732–20739.

Cox, J., and Mann, M. (2008). MaxQuant enables high peptide identification rates, individualized p.p.b.-range mass accuracies and proteome-wide protein quantification. Nat. Biotechnol. *26*, 1367–1372.

Denic, V., Quan, E.M., and Weissman, J.S. (2006). A luminal surveillance complex that selects misfolded glycoproteins for ER-associated degradation. Cell *126*, 349–359.

El Khouri, E., Le Pavec, G., Toledano, M.B., and Delaunay-Moisan, A. (2013). RNF185 is a novel E3 ligase of endoplasmic reticulum-associated degradation (ERAD) that targets cystic fibrosis transmembrane conductance regulator (CFTR). J. Biol. Chem. *288*, 31177–31191.

Fang, S., Ferrone, M., Yang, C., Jensen, J.P., Tiwari, S., and Weissman, A.M. (2001). The tumor autocrine motility factor receptor, gp78, is a ubiquitin protein ligase implicated in degradation from the endoplasmic reticulum. Proc. Natl. Acad. Sci. USA *98*, 14422–14427.

Flierman, D., Coleman, C.S., Pickart, C.M., Rapoport, T.A., and Chau, V. (2006). E2-25K mediates US11-triggered retro-translocation of MHC class I heavy chains in a permeabilized cell system. Proc. Natl. Acad. Sci. USA *103*, 11589–11594.

Foresti, O., Ruggiano, A., Hannibal-Bach, H.K., Ejsing, C.S., and Carvalho, P. (2013). Sterol homeostasis requires regulated degradation of squalene monooxygenase by the ubiquitin ligase Doa10/Teb4. eLife *2*, e00953.

Fueller, J., Herbst, K., Meurer, M., Gubicza, K., Kurtulmus, B., Knopf, J.D., Kirrmaier, D., Buchmuller, B.C., Pereira, G., Lemberg, M.K., et al. (2020). CRISPR-Cas12a-assisted PCR tagging of mammalian genes. J. Cell Biol. *219*, e201910210.

Gauss, R., Jarosch, E., Sommer, T., and Hirsch, C. (2006). A complex of Yos9p and the HRD ligase integrates endoplasmic reticulum quality control into the degradation machinery. Nat. Cell Biol. *8*, 849–854.

Guerriero, C.J., and Brodsky, J.L. (2012). The delicate balance between secreted protein folding and endoplasmic reticulum-associated degradation in human physiology. Physiol. Rev. *92*, 537–576.

Habeck, G., Ebner, F.A., Shimada-Kreft, H., and Kreft, S.G. (2015). The yeast ERAD-C ubiquitin ligase Doa10 recognizes an intramembrane degron. J. Cell Biol. *209*, 261–273.

Hampton, R.Y., Gardner, R.G., and Rine, J. (1996). Role of 26S proteasome and HRD genes in the degradation of 3-hydroxy-3-methylglutaryl-CoA reductase, an integral endoplasmic reticulum membrane protein. Mol. Biol. Cell *7*, 2029–2044.

Hart, T., Tong, A.H.Y., Chan, K., Van Leeuwen, J., Seetharaman, A., Aregger, M., Chandrashekhar, M., Hustedt, N., Seth, S., Noonan, A., et al. (2017). Evaluation and design of genome-wide CRISPR/SpCas9 knockout screens. G3 (Bethesda) *7*, 2719–2727.

Huang, E.Y., To, M., Tran, E., Dionisio, L.T.A., Cho, H.J., Baney, K.L.M., Pataki, C.I., and Olzmann, J.A. (2018). A VCP inhibitor substrate trapping approach (VISTA) enables proteomic profiling of endogenous ERAD substrates. Mol. Biol. Cell *29*, 1021–1030.

Hülsmann, J., Kravic, B., Weith, M., Gstaiger, M., Aebersold, R., Collins, B.C., and Meyer, H. (2018). AP-SWATH reveals direct involvement of VCP/p97 in integrated stress response signaling through facilitating CReP/PPP1R15B degradation. Mol. Cell. Proteomics *17*, 1295–1307.

Huyer, G., Piluek, W.F., Fansler, Z., Kreft, S.G., Hochstrasser, M., Brodsky, J.L., and Michaelis, S. (2004). Distinct machinery is required in Saccharomyces cerevisiae for the endoplasmic reticulum-associated degradation of a multispanning membrane protein and a soluble luminal protein. J. Biol. Chem. *279*, 38369–38378.

Hwang, J., Walczak, C.P., Shaler, T.A., Olzmann, J.A., Zhang, L., Elias, J.E., and Kopito, R.R. (2017). Characterization of protein complexes of the endoplasmic reticulum-associated degradation E3 ubiquitin ligase Hrd1. J. Biol. Chem. *292*, 9104–9116.

Jiang, L.-L., Zhu, B., Zhao, Y., Li, X., Liu, T., Pina-Crespo, J., Zhou, L., Xu, W., Rodriguez, M.J., Yu, H., et al. (2019). Membralin deficiency dysregulates astrocytic glutamate homeostasis leading to ALS-like impairment. J. Clin. Invest. *129*, 3103–3120.

Jo, Y., Sguigna, P.V., and DeBose-Boyd, R.A. (2011). Membrane-associated ubiquitin ligase complex containing gp78 mediates sterol-accelerated degradation of 3-hydroxy-3-methylglutaryl-coenzyme A reductase. J. Biol. Chem. *286*, 15022–15031.

Kaneko, M., Iwase, I., Yamasaki, Y., Takai, T., Wu, Y., Kanemoto, S., Matsuhisa, K., Asada, R., Okuma, Y., Watanabe, T., et al. (2016). Genome-wide identification and gene expression profiling of ubiquitin ligases for endoplasmic reticulum protein degradation. Sci. Rep. *6*, 30955.

Leto, D.E., Morgens, D.W., Zhang, L., Walczak, C.P., Elias, J.E., Bassik, M.C., and Kopito, R.R. (2019). Genome-wide CRISPR analysis identifies substrate-specific conjugation modules in ER-associated degradation. Mol. Cell *73*, 377–389.e11.

CellPress
OPEN ACCESS

Li, W., Bengtson, M.H., Ulbrich, A., Matsuda, A., Reddy, V.A., Orth, A., Chanda, S.K., Batalov, S., and Joazeiro, C.A.P. (2008). Genome-wide and functional annotation of human E3 ubiquitin ligases identifies MULAN, a mitochondrial E3 that regulates the organelle's dynamics and signaling. PLoS ONE *3*, e1487.

Li, W., Xu, H., Xiao, T., Cong, L., Love, M.I., Zhang, F., Irizarry, R.A., Liu, J.S., Brown, M., and Liu, X.S. (2014). MAGeCK enables robust identification of essential genes from genome-scale CRISPR/Cas9 knockout screens. Genome Biol. *15*, 554.

Li, W., Köster, J., Xu, H., Chen, C.-H., Xiao, T., Liu, J.S., Brown, M., and Liu, X.S. (2015). Quality control, modeling, and visualization of CRISPR screens with MAGeCK-VISPR. Genome Biol. *16*, 281.

Mehrtash, A.B., and Hochstrasser, M. (2019). Ubiquitin-dependent protein degradation at the endoplasmic reticulum and nuclear envelope. Semin. Cell Dev. Biol. *93*, 111–124.

Michel, M.A., Elliott, P.R., Swatek, K.N., Simicek, M., Pruneda, J.N., Wagstaff, J.L., Freund, S.M.V., and Komander, D. (2015). Assembly and specific recognition of k29- and k33-linked polyubiquitin. Mol. Cell *58*, 95–109.

Monk, B.C., Tomasiak, T.M., Keniya, M.V., Huschmann, F.U., Tyndall, J.D.A., O'Connell, J.D., 3rd, Cannon, R.D., McDonald, J.G., Rodriguez, A., Finer-Moore, J.S., and Stroud, R.M. (2014). Architecture of a single membrane spanning cytochrome P450 suggests constraints that orient the catalytic domain relative to a bilayer. Proc. Natl. Acad. Sci. USA *111*, 3865–3870.

Morito, D., Hirao, K., Oda, Y., Hosokawa, N., Tokunaga, F., Cyr, D.M., Tanaka, K., Iwai, K., and Nagata, K. (2008). Gp78 cooperates with RMA1 in endoplasmic reticulum-associated degradation of CFTRDeltaF508. Mol. Biol. Cell *19*, 1328–1336.

Natarajan, N., Foresti, O., Wendrich, K., Stein, A., and Carvalho, P. (2020). Quality control of protein complex assembly by a transmembrane recognition factor. Mol. Cell *77*, 108–119.e9.

Neutzner, A., Neutzner, M., Benischke, A.-S., Ryu, S.-W., Frank, S., Youle, R.J., and Karbowski, M. (2011). A systematic search for endoplasmic reticulum (ER) membrane-associated RING finger proteins identifies Nixin/ZNRF4 as a regulator of calnexin stability and ER homeostasis. J. Biol. Chem. *286*, 8633–8643.

Qiu, D., Wang, Q., Wang, Z., Chen, J., Yan, D., Zhou, Y., Li, A., Zhang, R., Wang, S., and Zhou, J. (2018). RNF185 modulates JWA ubiquitination and promotes gastric cancer metastasis. Biochim. Biophys. Acta Mol. Basis Dis. *1864* (5 Pt A), 1552–1561.

Ramachandran, S., Osterhaus, S.R., Parekh, K.R., Jacobi, A.M., Behlke, M.A., and McCray, P.B., Jr. (2016). SYVN1, NEDD8, and FBXO2 proteins regulate ΔF508 cystic fibrosis transmembrane conductance regulator (CFTR) ubiquitin-mediated proteasomal degradation. J. Biol. Chem. *291*, 25489–25504.

Ruggiano, A., Foresti, O., and Carvalho, P. (2014). Quality control: ER-associated degradation: protein quality control and beyond. J. Cell Biol. *204*, 869–879.

Sato, Y., Koshioka, S., Kirino, Y., Kamimoto, T., Kawazoe, K., Abe, S., et al. (2011). Role of dipeptidyl peptidase IV (DPP4) in the development of dyslipidemia: DPP4 contributes to the steroid metabolism pathway. Life Sci. *88*, 43–49.

Stefanovic-Barrett, S., Dickson, A.S., Burr, S.P., Williamson, J.C., Lobb, I.T., van den Boomen, D.J., Lehner, P.J., and Nathan, J.A. (2018). MARCH6 and TRC8 facilitate the quality control of cytosolic and tail-anchored proteins. EMBO Rep. *19*, e45603.

Swanson, R., Locher, M., and Hochstrasser, M. (2001). A conserved ubiquitin ligase of the nuclear envelope/endoplasmic reticulum that functions in both ER-associated and Matalpha2 repressor degradation. Genes Dev. *15*, 2660–2674.

Tyanova, S., Temu, T., and Cox, J. (2016a). The MaxQuant computational platform for mass spectrometry-based shotgun proteomics. Nat. Protoc. *11*, 2301–2319.

Tyanova, S., Temu, T., Sinitcyn, P., Carlson, A., Hein, M.Y., Geiger, T., Mann, M., and Cox, J. (2016b). The Perseus computational platform for comprehensive analysis of (prote)omics data. Nat. Methods *13*, 731–740.

van de Weijer, M.L., Bassik, M.C., Luteijn, R.D., Voorburg, C.M., Lohuis, M.A.M., Kremmer, E., Hoeben, R.C., LeProust, E.M., Chen, S., Hoelen, H., et al. (2014). A high-coverage shRNA screen identifies TMEM129 as an E3 ligase involved in ER-associated protein degradation. Nat. Commun. *5*, 3832.

van de Weijer, M.L., Schuren, A.B.C., van den Boomen, D.J.H., Mulder, A., Claas, F.H.J., Lehner, P.J., et al. (2017). Multiple E2 ubiquitin-conjugating enzymes regulate human cytomegalovirus US2-mediated immunoreceptor downregulation. J Cell Sci *130*, 2883–2892.

Vashist, S., and Ng, D.T.W. (2004). Misfolded proteins are sorted by a sequential checkpoint mechanism of ER quality control. J. Cell Biol. *165*, 41–52.

Walter, J., Urban, J., Volkwein, C., and Sommer, T. (2001). Sec61p-independent degradation of the tail-anchored ER membrane protein Ubc6p. EMBO J. *20*, 3124–3131.

Wang, Y., Pearce, M.M.P., Sliter, D.A., Olzmann, J.A., Christianson, J.C., Kopito, R.R., Boeckmann, S., Gagen, C., Leichner, G.S., Roitelman, J., and Wojcikiewicz, R.J. (2009). SPFH1 and SPFH2 mediate the ubiquitination and degradation of inositol 1,4,5-trisphosphate receptors in muscarinic receptor-expressing HeLa cells. Biochim. Biophys. Acta *1793*, 1710–1718.

Wiśniewski, J.R., Zougman, A., Nagaraj, N., and Mann, M. (2009). Universal sample preparation method for proteome analysis. Nat. Methods *6*, 359–362.

Wu, X., and Rapoport, T.A. (2018). Mechanistic insights into ER-associated protein degradation. Curr. Opin. Cell Biol. *53*, 22–28.

Xie, W., Kanehara, K., Sayeed, A., and Ng, D.T.W. (2009). Intrinsic conformational determinants signal protein misfolding to the Hrd1/Htm1 endoplasmic reticulum-associated degradation system. Mol. Biol. Cell *20*, 3317–3329.

Yang, H., Takagi, H., Konishi, Y., Ageta, H., Ikegami, K., Yao, I., Sato, S., Hatanaka, K., Inokuchi, K., Seog, D.-H., and Setou, M. (2008). Transmembrane and ubiquitin-like domain-containing protein 1 (Tmub1/HOPS) facilitates surface expression of GluR2-containing AMPA receptors. PLoS ONE *3*, e2809.

Yang, B., Qu, M., Wang, R., Chatterton, J.E., Liu, X.-B., Zhu, B., Narisawa, S., Millan, J.L., Nakanishi, N., Swoboda, K., et al. (2015). The critical role of membralin in postnatal motor neuron survival and disease. eLife *4*, e06500.

Yau, R.G., Doerner, K., Castellanos, E.R., Haakonsen, D.L., Werner, A., Wang, N., Yang, X.W., Martinez-Martin, N., Matsumoto, M.L., Dixit, V.M., and Rape, M. (2017). Assembly and function of heterotypic ubiquitin chains in cell-cycle and protein quality control. Cell *171*, 918–933.e20.

Younger, J.M., Chen, L., Ren, H.-Y., Rosser, M.F.N., Turnbull, E.L., Fan, C.-Y., Patterson, C., and Cyr, D.M. (2006). Sequential quality-control checkpoints triage misfolded cystic fibrosis transmembrane conductance regulator. Cell *126*, 571–582.

Zhu, B., Jiang, L., Huang, T., Zhao, Y., Liu, T., Zhong, Y., Li, X., Campos, A., Pomeroy, K., Masliah, E., et al. (2017). ER-associated degradation regulates Alzheimer's amyloid pathology and memory function by modulating γ-secretase activity. Nat. Commun. *8*, 1472.

STAR★METHODS

KEY RESOURCES TABLE

REAGENT or RESOURCE	SOURCE	IDENTIFIER
Antibodies		
Rabbit Monoclonal anti-VCP clone EPR3307(2)	Abcam	Cat# ab109240, RRID:AB_10862588
Rabbit Polyclonal anti-SPP C-term	Abcam	ab190253
Rabbit Monoclonal anti-Calnexin N-term clone EPR3632	Abcam	Cat# ab92573, RRID:AB_10563673
Rabbit Monoclonal anti-RNF185 clone EPR14070-94	Abcam	ab181999
Rabbit Monoclonal anti-Erlin2 clone EPR8089	Abcam	Cat# ab128924, RRID:AB_11150974
Rabbit Polyclonal anti-UBE3C	Abcam	ab226173
Rabbit Monoclonal anti-TMUB1 clone EPR14066	Abcam	ab180586
Rabbit Polyclonal anti-SEL1L	Abcam	Cat# ab78298, RRID:AB_2285813
Rabbit Monoclonal anti-BiP clone EPR4040(2)	Abcam	Cat# ab108613, RRID:AB_10859806
Rabbit Polyclonal anti-Membralin/TMEM259	Atlas Antibodies	Cat# HPA042669, RRID:AB_10794916
Rabbit Polyclonal anti-TMEM41B	Atlas Antibodies	Cat# HPA014946, RRID:AB_2205037
Mouse Monoclonal anti-VCP/p97 clone Clone18/VCP	BD Biosciences	Cat# 612183, RRID:AB_399554
Rabbit Monoclonal anti-HRD1 clone D3O2A	Cell Signaling Technology	Cat# 14773, RRID:AB_2798607
Rabbit Monoclonal anti-HIP2/UBE2K clone D27C4	Cell Signaling Technology	Cat# 8226, RRID:AB_10827650
Rabbit Polyclonal anti-Ub	Cell Signaling Technology	Cat# 3933, RRID:AB_2180538
Rabbit Monoclonal anti-VMP1 clone D6N4G	Cell Signaling Technology	Cat# 12978, RRID:AB_2798077
Rabbit Polyclonal anti-mNeonGreen	Cell Signaling Technology	Cat# 53061, RRID:AB_2799426
Rabbit Polyclonal anti-LC3B	Cell Signaling Technology	Cat# 2775, RRID:AB_915950
Rabbit Monoclonal anti-PDI clone C81H6	Cell Signaling Technology	Cat# 3501, RRID:AB_2156433
Mouse Monoclonal anti-CHOP clone L63F7	Cell Signaling Technology	Cat# 2895, RRID:AB_2089254
Rabbit Polyclonal anti-AMFR/gp78	Cell Signaling Technology	Cat# 9590, RRID:AB_10860080
Mouse Monoclonal anti-BAP31 clone A1/182	Enzo Life Sciences	Cat# ALX-804-601-C100, RRID:AB_2050797
Rabbit Polyclonal anti-TMUB2	ProteinTech	28044-1-AP
Rabbit Polyclonal anti-CYP51A1	ProteinTech	Cat# 13431-1-AP, RRID:AB_2088571
Mouse Monoclonal anti-GAPDH clone 1E6D9	ProteinTech	Cat# 60004-1-Ig, RRID:AB_2107436
Rabbit Polyclonal anti-CKAP4 (Climp63)	ProteinTech	Cat# 16686-1-AP, RRID:AB_2276275
Rabbit Polyclonal anti-Erlin1	ProteinTech	Cat# 17311-1-AP, RRID:AB_2098590
Rat Monoclonal anti-HA clone 3F10	Roche	Cat# 11867423001, RRID:AB_390918
Rabbit Monoclonal anti-RNF5 clone 22B3	Santa Cruz Biotechnology	Cat# sc-81716, RRID:AB_2238618
Rat Monoclonal anti-Tubulin clone YOL1/34	Santa Cruz Biotechnology	Cat# sc-53030, RRID:AB_2272440
Mouse Monoclonal anti-FLAG-HRP clone M2	Sigma-Aldrich	Cat# A8592, RRID:AB_439702

(*Continued on next page*)

Continued

REAGENT or RESOURCE	SOURCE	IDENTIFIER
Peroxidase AffiniPure F(ab')$_2$ Fragment Donkey Anti-Rabbit IgG (H+L)	Jackson ImmunoResearch	Labs Cat# 711-036-152, RRID:AB_2340590
Peroxidase AffiniPure Donkey Anti-Rat IgG (H+L)	Jackson ImmunoResearch	Cat# 712-035-150, RRID:AB_2340638
Peroxidase AffiniPure Donkey Anti-Mouse IgG (H+L)	Jackson ImmunoResearch	Cat# 715-035-150, RRID:AB_2340770
Peroxidase IgG Fraction Monoclonal Mouse Anti-Rabbit IgG, light chain specific	Jackson ImmunoResearch	Cat# 211-032-171, RRID:AB_2339149
Peroxidase AffiniPure Goat Anti-Rat IgG, light chain specific	Jackson ImmunoResearch	Cat# 112-035-175, RRID:AB_2338140
Peroxidase AffiniPure Goat Anti-Mouse IgG, light chain specific	Jackson ImmunoResearch	Cat# 115-035-174, RRID:AB_2338512
Alexa Fluor 568 goat anti-mouse IgG (H+L) secondary antibody	Thermo Fisher Scientific	Cat# A-11004, RRID:AB_2534072
Chemicals, Peptides, and Recombinant Proteins		
DMEM medium	Sigma-Aldrich	Cat# D6429
L-Glutamine (200 mM)	GIBCO (Thermo Fisher Scientific)	Cat# 25030024
Penicillin-Streptomycin (10,000 U/mL)	GIBCO (Thermo Fisher Scientific)	Cat# 15140122
10% FBS	GIBCO (Thermo Fisher Scientific)	Cat# 26140079
Zeocin	Invitrogen (Thermo Fisher Scientific)	Cat# R25001
Puromycin	GIBCO (Thermo Fisher Scientific)	Cat# A1113803
Tetracycline	Sigma-Aldrich	Cat# T7660
Cycloheximide	Sigma-Aldrich	Cat# C7698
TransIT LT1	Mirus Bio LLC	Cat# MIR 2305
OptiMEM	GIBCO (Thermo Fisher Scientific)	Cat# 31985062
3xFLAG peptide	Sigma-Aldrich	Cat# F4799
cOmplete EDTA-free protease inhibitor cocktail	Roche	Cat# 5056489001
Benzonase	Sigma-Aldrich	Cat# E1014
N-Ethylmaleimide (NEM)	Sigma-Aldrich	Cat# E3876
1,4-Dithiothreitol (DTT)	Sigma-Aldrich	Cat# D9779
PMSF	Roche	Cat# 11359061001
Pepstatin A	Sigma-Aldrich	Cat# P5318
DNase I	Roche	Cat# 11284932001
DMNG	Anatrace	Cat# NG322
DDM	Anatrace	Cat# D310
CHS	Anatrace	Cat# CH210
Tunicamycin	Cell Signaling Technology	Cat# 12819S
Thapsigargin	Cell Signaling Technology	Cat# 12758S
CB-5083	Selleckchem	Cat# S8101
dNTP mixture	Takara	Cat# 4030
First Strand buffer	Invitrogen (Thermo Fisher Scientific)	Cat# 18080093
RNaseOUT	Invitrogen (Thermo Fisher Scientific)	Cat# 10777019
Fluoromount-G (with DAPI)	Invitrogen (Thermo Fisher Scientific)	Cat# 00-4959-52
Critical Commercial Assays		
FLAG-M2 Magnetic Beads	Sigma-Aldrich	Cat# M8823, RRID:AB_2637089
Pierce Anti-HA Magnetic Beads	Thermo Fisher Scientific	Cat# 88837
Pierce Streptavidin Magnetic Beads	Thermo Fisher Scientific	Cat# 88817
mNeonGreen-Trap Magnetic Agarose	Chromotek	Cat# ntma-20

(*Continued on next page*)

Continued

REAGENT or RESOURCE	SOURCE	IDENTIFIER
QIAGEN BloodMaxi kit	QIAGEN	Cat# 51194
QIAGEN BloodMini kit	QIAGEN	Cat# 51106
NEBNext® Ultra II Q5® Master Mix	New England Biolabs	Cat# M0544L
SuperScriptIII	Invitrogen (Thermo Fisher Scientific)	Cat# 18080093
SensiFAST SYBR® No-ROX kit	Bioline	Cat# BIO-98020
Monarch® Total RNA Miniprep Kit	New England Biolabs	Cat# T2010S
Western Lightning ECL Pro	Perkin Elmer	Cat# NEL121001EA
Deposited Data		
Genome-wide CRISPR-Cas9 screen	This study; ENA Data Set	Data S2; PRJEB37897
Proteomics	This study; PRIDE Data Set	Data S3, S4, and S5; PXD018517
Original western blot images	This study; Mendeley Data Set	https://doi.org/10.17632/ftrm9sc5m9.1
Experimental Models: Cell Lines		
Flp-In T-REx 293 Cell Line	Invitrogen (Thermo Fisher Scientific)	Cat# R78007
Lenti-X 293T Cell Line	Clontech (Takara Bio)	Cat# 632180
HeLa Cell Line	ATCC	ATCC® CCL-2
Oligonucleotides		
Focused sgRNA library targeting E2s and E3s	This study; (van de Weijer et al., 2017)	Data S1
Z07 MBRL-mNeonGreen PCR tagging M1: GCTTCTGACACAACTCCCCTGGGGGC TGCGGTAGGCGGGCCTAGCCCGGCC TCCATGGCCCCAACGGAGGCGCCC TCGGAGGTGGGGTCCTCAGGTGGAG GAGGTAGTG	Integrated DNA Technologies (IDT)	N/A
Z07 MBRL-mNeonGreen PCR tagging M2: GGCAGTCCCAGAGGAAGGA GGTGGCTGGCCTCCCCCACCCC CACGGGCTCGGGAAAAAAAACAG GCCCAGCCAGCAGGGGTATCTAC ACTTAGTAGAAATTAGCTAGCTG CATCGGTACC	Integrated DNA Technologies (IDT)	N/A
Z07 HRD1-mNeonGreen PCR tagging M1: GGCACAGAGGAGATGCCTGA GGATGGAGAGCCCGATGCAGCAG AGCTCCGCCGGCGCCGCCTGCAG AAGCTGGAGTCTCCTGTTGCCC ACTCAGGTGGAGGAGGTAGTG	Integrated DNA Technologies (IDT)	N/A
Z07 HRD1-mNeonGreen PCR tagging M2: TGTTCCAGCGAGGGCTGCTCAA AAGAGCAGAGGCTGGGGCTGGGCTG GGGCAGTGAAAAAAGCAGTGTCAGT GGGCAACAGATCTACACTTAGTAGA AATTAGCTAGCTGCATCGGTACC	Integrated DNA Technologies (IDT)	N/A
OligodT-18-mer	Integrated DNA Technologies (IDT)	Cat# 51-01-15-07
qPCR Human *BiP* primer pair: 5′-GGATCATCAACGAGCCTACG-3′ (forward); 5′-CACCCAGGTCAAAC ACCAG-3′ (reverse)	Integrated DNA Technologies (IDT);	N/A
qPCR Human *RNF185* primer pair: 5′-CTGTCACGCCTCTTCCTATTTGT-3′ (forward); 5′-GCCCAGCATTAG GCAATCAG-3′(reverse)	Integrated DNA Technologies (IDT); (El Khouri et al., 2013)	N/A

(*Continued on next page*)

Continued

REAGENT or RESOURCE	SOURCE	IDENTIFIER
qPCR Human *MBRL* primer pair: 5′-TCACTACCGCTTCAATGGGCAG-3′ (forward); 5′-CTGAAGCAGCA TCTCCTGGATG-3′(reverse)	Integrated DNA Technologies (IDT); OriGene pre-design primers	N/A
qPCR Human *CYP51A1* primer pair: 5′-CTACAGTCGCCTGACAACAC-3′ (forward); 5′-CCACTTTCTCC CCAACTCTC-3′ (reverse)	Integrated DNA Technologies (IDT); (Sato et al., 2011)	N/A
qPCR Human *GAPDH* primer pair: 5′-AACCTGCCAAATATGATGAC-3′ (forward); 5′-AGGAAATGAGCT TGACAAAG-3′(reverse)	Integrated DNA Technologies (IDT)	N/A
Recombinant DNA		
pcDNA5-FRT-TO plasmid	Thermo Fisher Scientific	V652020
Lentiviral cDNA expression vectors	(van de Weijer et al., 2014)	N/A
PCR-tagging plasmid Z07-mNeongreen	(Fueller et al., 2020)	Addgene Plasmid #124790
Toronto KnockOut (TKO) CRISPR Library - Version 3	(Hart et al., 2017)	Addgene #90294
Software and Algorithms		
MaxQuant, version 1.6.3.4	MaxQuant	https://www.maxquant.org/
Perseus software, version 1.5.5.3	MaxQuant	https://maxquant.net/perseus/
SlideBook 6	3i - Intelligent Imaging Innovations	https://www.intelligent-imaging.com/slidebook
Image studio software Li-Cor	Li-Cor	https://www.licor.com/bio/image-studio-lite/
FlowJo 10.4	FlowJo, LLC	https://www.flowjo.com/
GraphPad Prism	GraphPad	https://www.graphpad.com/scientific-software/prism/
MAGeCK	(Li et al., 2014) (Li et al., 2015)	N/A

RESOURCE AVAILABILITY

Lead Contact

Further information and requests for reagents may be directed to and will be fulfilled by the Lead Contact, Pedro Carvalho (pedro.carvalho@path.ox.ac.uk).

Materials Availability

All unique reagents generated in this study are available from the Lead Contact without restriction.

Data and Code Availability

The raw reads dataset generated during this study is available at ENA (https://www.ebi.ac.uk/ena/data/view/PRJEB37897).

The raw proteomics dataset generated during this study is available at PRIDE (https://www.ebi.ac.uk/pride/archive/projects/PXD018517).

The raw fluorescence microscopy and western blotting data generated during this study are available at Mendeley Data (https://doi.org/10.17632/ftrm9sc5m9.1).

The published article includes all processed data generated or analyzed during this study as Supplemental Information.

EXPERIMENTAL MODEL AND SUBJECT DETAILS

Flp-In T-REx HEK293 cells were obtained from Invitrogen (Thermo Fischer Scientific). Flp-In T-REx HEK293 clones were established using manufacturer's guidelines. HeLa cells were obtained from ATCC (American Type Culture Collection). 293T Lenti-X virus

packaging cells were obtained from Takara Clontech. Cells were grown at 37°C 5% CO_2 in DMEM medium (Sigma-Aldrich) supplemented with L-Glutamine (2 mM; GIBCO), Penicillin-Streptomycin (10 Units/mL; GIBCO) and 10% FCS (GIBCO).

METHOD DETAILS

Lentivirus production

For individual gene infections using lentiviruses, virus was produced in 24-well plates using TransIT LT1 (Mirus Bio LLC) and second-generation packaging vectors according to standard lentiviral production protocols.

Plasmids

The pcDNA5-FRT-TO plasmid was obtained from Invitrogen. cDNAs or sgRNAs, respectively for protein overexpression and gene deletions, were cloned in a dual promoter lentiviral vector, as described previously (van de Weijer et al., 2014).

Genome-wide CRISPR/Cas9 screen

The TKOv3 CRISPR/Cas9 library was a gift from Jason Moffat (Addgene #90294). The sgRNA library and 2nd generation lentiviral packaging plasmids were transfected into 293T cells. After 72 h, lentivirus was harvested and infected into 125x10^6 Flp-In T-REx 293 CYP51A1TM-sfGFP-3xHA cells at an MOI of 0.3 to achieve a 250-fold coverage of the library after selection. Cells were grown for 48 h and then selected with 2 μg/mL puromycin (GIBCO) for 72 h. Cells were then split into two technical replicates. 24 h prior to sorting, 100 ng/mL tetracycline (Sigma-Aldrich) was added to the cell medium to induce the expression of the ERAD reporter. 2% of the brightest GFP (GFP^{high}) cells from 25x10^6 cells was collected using a BD FACSAria3 and a Beckman Coulter MoFlo Astrios. Genomic DNA was extracted from each cell population using a QIAGEN BloodMaxi kit (for reference samples) or BloodMini kit (for sorted GFP^{high} samples) according to the manufacturer's protocol. SgRNAs were PCR amplified from the entire isolated genomic DNA using NEBNext® Ultra II Q5® Master Mix (NEB) and the primers v2.1-F1 and v2.1-R1, according to the TKOv3 protocol. PCR reactions were pooled again, after which a second PCR was performed to attach indices and sequencing adapters using the primers i5 and i7. The PCR reaction was loaded onto a 2% agarose gel, the 200bp band excised and purified using a GeneJet PCR Purification kit (Thermo Fischer Scientific). Libraries were analyzed by deep sequencing on an Illumina HiSeq4000. Gene rankings were generated using the MAGeCK algorithm (Li et al., 2014, 2015).

Generation of CRISPR/Cas9-mediated knockout cells

For CRISPR/Cas9-mediated knockouts, cell lines were transfected using Mirus LT-1 using manufacturer's protocol. On the next day, cells were selected using Puromycin (2 μg/mL; GIBCO). After 48 hours of selection, medium was replaced with standard medium. To generate KO clones, cells were single-cell sorted using a BD FACSAria3 or a Beckman Coulter MoFlo Astrios. Knockout status of the clones was confirmed via flow cytometry and/or western blotting.

Endogenous tagging

MBRL and HRD1 were endogenously tagged with mNeonGreen at their C terminus in HeLa cells using the PCR-tagging technique (Fueller et al., 2020). Cells were selected using Zeocin (100 μg/mL; Invitrogen) for 7 days, after which mNeonGreen-positive cells were single-cell sorted using a BD FACSAria3 or a Beckman Coulter MoFlo Astrios. Correct tagging of the genes was assessed by a gene-specific sgRNA, which abrogated all fluorescence, and by western blotting with a gene product-specific antibody.

Co-immunoprecipitations

Cells were lysed in 1% DMNG (Anatrace) lysis buffer (50 mM Tris–HCl pH 7.5, 150 mM NaCl) containing cOmplete protease inhibitor cocktail (Roche). Lysates were rotated for 60 min at 4°C. Cell debris and nuclei were pelleted at 13,000 g for 20 min at 4°C. Post-nuclear supernatants were incubated for 2 h with mNeonGreen-Trap magnetic beads (Chromotek) or FLAG-M2 magnetic beads (Sigma-Aldrich). After four washes in 0.1% DMNG washing buffer, proteins were eluted in 1x sample buffer for 30 min at 37°C or using 3xFLAG peptide (500 ng/uL; Sigma-Aldrich) for 30 min on ice, respectively. In the case of elution using FLAG-peptide, the eluate was transferred to a new tube and subsequently denatured by adding Laemmli sample buffer containing DTT. Immunoblotting was performed as described below.

Substrate ubiquitination experiments

Cells at around 80% confluency in a 10 cm dish were lysed in RIPA buffer (50 mM Tris-HCl pH 7.5, 150 mM NaCl, 1% Triton X-100, 0.5% Sodium Deoxycholate, 0.1% SDS) containing NEM (20 mM) and cOmplete protease inhibitor cocktail (Roche). Lysates were rotated for 60 min at 4°C. Cell debris and nuclei were pelleted at 13,000 g for 20 min at 4°C. Post-nuclear supernatants were incubated for 2 h with anti-HA magnetic beads (Thermo Fischer Scientific). After four washes in RIPA buffer, proteins were eluted in sample buffer. The eluate was transferred to a new tube and subsequently reduced using DTT. Immunoblotting was performed as described below.

Translation shut-off experiments

Cells were incubated with cycloheximide (50 μg/mL) for the indicated time points, after which cells were lysed in 1x sample buffer containing Benzonase (Sigma-Aldrich), cOmplete protease inhibitor cocktail (Roche), and DTT. Lysates were incubated for 30 min at 37°C, after which proteins were separated by SDS-PAGE. Immunoblotting was performed as described below.

Immunoblotting

Samples were incubated at 37°C for 15 min, separated by SDS–PAGE (Bio-Rad) and proteins were transferred to PVDF membranes (Bio-Rad). Membranes were probed with indicated antibodies. Reactive bands were detected by ECL (Western Lightning ECL Pro, Perkin Elmer), and visualized using an Amersham Imager 600 (GE Healthcare Life Sciences).

Data quantification was performed using Image Studio software (Li-Cor) and graphs were plotted in GraphPad Prism. Representative images of three independent experiment are shown.

Size-exclusion

Endogenously tagged MBRL-mNG HeLa cells were expanded to 3x 15 cm dishes. Cells were harvested and resuspended in 60 mL of chilled PBS (Sigma-Aldrich) supplemented with a cOmplete protease inhibitor cocktail table (Roche), 1 mM EDTA, 1 mM PMSF (Roche), 1.5 μM pepstatin A (Sigma-Aldrich) and 20 μg/mL of DNase I (Roche). Lysis was performed by passing the cells 5 times through an Avestin EmulsiFlex-C5 at 12,000 PSI of backpressure. The lysate was centrifuged at 12,000 g for 20 min at 4°C and the supernatant collected. Membranes were pelleted by centrifugation at 100,000 g for 60 min at 4°C. Pelleted membranes were resuspended in 2 mL of resuspension buffer containing 100 mM Tris-HCl (pH 7.5), 150 mM NaCl, 1% DDM (Anatrace), 0.1% CHS (Anatrace), 1 mM EDTA, 1 mM PMSF and 1.5 μM pepstatin A. Membranes were solubilised for 2 h on a rotator at 4°C and insoluble material pelleted by centrifugation at 100,000 g for 45 min at 4°C. Solubilised material was applied to a 24 mL Superose 6 10/300 GL column (GE Healthcare Life Sciences) equilibrated with 2 column volumes of 50 mM Tris-HCl (pH 7.4), 150 mM NaCl, 0.03% DDM and 0.003% CHS. Elutions were fractioned by 1.0 mL and aliquots run by SDS-PAGE for western blotting.

Mass Spectrometry

Protein samples were digested as described (Wiśniewski et al., 2009). Peptide samples from 3xFLAG- and mNG-tagged immunoprecipitates were separated by nano-flow reversed-phase liquid chromatography coupled to Q Exactive and Q Exactive HF Hybrid Quadrupole-Orbitrap instrument (Thermo Fisher Scientific), respectively. The mass spectrometers were operated in a data-dependent mode; the 10 or 12 most intense precursor ions were submitted to fragmentation in the mNG and 3xFLAG samples, respectively. MS/MS spectra were searched against the Uniprot human reference proteome database (UP000005640, retrieved 2019-07-02) using MaxQuant, version 1.6.3.4 (Cox and Mann, 2008; Tyanova et al., 2016a). False discovery rate for both protein and peptide matches was set at 1%. Data analysis was performed using Perseus software, version 1.5.5.3 (Tyanova et al., 2016b).

Fluorescence microscopy

Flp-In T-REx 293 cells stably expressing Tetracycline-inducible sfGFP-fusion constructs were seeded onto an 8-well Lab-Tek® II CC2 Chamber Slide system (Thermo Fisher Scientific, 154941) at a density of 15,000 cells/well. On the next day, sfGFP-fusion constructs were induced with 100 ng/ml Tetracycline for 20 hours. In the last 4 h, cells were incubated with 2.5 μM CB-5083. Cells were then fixed in 4% Paraformaldehyde in PBS for 10 min, followed by three washes with PBS. Next, cells were incubated with 10 mM Glycine (in PBS) for 5 min followed by three washes with PBS. Prior to immunostaining, cells were incubated in blocking buffer (BB; 1% BSA and 0.1% Saponin in PBS) for 30 min. Cells were then incubated with mouse anti-human Bap31 monoclonal antibody (Enzo Life Sciences) at 1:300 dilution in BB for 1 h, followed by three washes with BB. Next, cells were incubated with Alexa Fluor 568 goat anti-mouse IgG (H+L) secondary antibody (Thermo Fisher Scientific) at 1:400 dilution in BB for 1 h, followed by three washes with BB and three washes with PBS. After removing the PBS, Fluoromount-G (with DAPI; Invitrogen) was added to each well. Next, the chamber was carefully peeled off the slide and covered with a coverslip. The mounting media was allowed to solidify for at least 2 h and sealed with nail polish. Slides were kept at 4°C until imaging on a Zeiss Axio Observer Z1 with a CMOS camera (Hamamatsu ORCA-Flash4.0) controlled by 3i Slidebook6.0 software. A 100 × 1.4 NA Plan Apochromat oil immersion objective was used.

Membrane isolation

Cells were grown in a 15 cm dish to 80%–90% confluency. Cells were washed once with PBS and once with hypotonic buffer (10 mM HEPES-KOH pH 7.4, 1.5 mM $MgCl_2$, 10 mM KCl, 0.5 mM DTT, 10 μg/mL PMSF, cOmplete EDTA-free protease inhibitor cocktail), after which cells were detached in 4 mL hypotonic buffer using a cell scraper. Cells were incubated for 15 min on ice and afterward mechanically lysed using a douncer. Unlysed cells and debris were pelleted at 1,000 g for 5 min at 4°C twice. The resulting crude membranes were pelleted at 100,000 g for 30 min at 4°C. The membrane pellet was resuspended in 1 mL RM buffer (250 mM sucrose, 50 mM HEPES-KOH pH 7.4, 50 mM KOAc, 2 mM $Mg(OAc)_2$, 1 mM DTT), after which 0.5 mL of a 1.5 M KOAc 150 mM EDTA solution was slowly added. Membranes were incubated for 15 minutes on ice. The membranes were loaded onto a 1.5 mL high-salt sucrose cushion (500 mM sucrose, 50 mM HEPES-KOH pH 7.4, 500 mM KOAc, 5 mM $Mg(OAc)_2$) and pelleted at 100,000 g for 60 min at 4°C. The resulting membrane pellet was resuspended in freshly prepared ice-cold sodium carbonate solution

(125 mM sucrose, 100 mM Na_2CO_3). Membranes were pelleted at 100,000 g for 60 min at 4°C. The membrane pellet was then solubilized in 1% SDS-TBS buffer.

Quantitative PCR analysis

For analysis of *RNF185, TMEM259 (Membralin), CYP51A1* and *GRP78 (BiP)* gene expression upon unfolded protein response (UPR) induction, HEK293 T-REx Flp-In parental, RNF185 KO and Membralin KO cells were grown overnight in 6-well plates to achieve confluency of about 80%. Next, cells were treated with either DMSO or 2 μg/mL Tunicamycin (Cell Signaling) for 12 hours. Treated cells were harvested by trypsinisation and cell pellets were collected by centrifugation (5 min, 500 g) and washed once with PBS. Cell pellets were subjected to RNA extraction using the Monarch® Total RNA Miniprep Kit (NEB) according to the manufacturer's instructions. RNA purity and concentration was determined using a NanoPhotometer® (Implen) and 500 ng of RNA was subsequently used for cDNA synthesis reaction. For each sample, 5 μl of RNA (100 ng/μL) was mixed with 1 μL of 50 μM OligodT-18-mer (IDT), 4 μL of 2.5 mM dNTP mixture (Takara) and 3 μL of Diethyl Pyrocarbonate (DEPC)-treated water and subjected to denaturation/oligodT binding using a thermal cycler (Step 1, 65°C for 5 min; and Step 2, 4°C for 10 min). 7 μL of reverse transcriptase mastermix (4 μL of 5x First Strand buffer (Invitrogen), 1 μL of 0.1 M DTT, 1 μL of RNaseOUT (Invitrogen) and 1 μL of SuperScriptIII (Invitrogen) was added to each RNA/OligodT mixture and mixed by gentle pipetting. cDNA synthesis was performed in a thermal cycler (Step 1, 25°C for 5 min; Step 2, 50°C for 60 min; and Step 3, 70°C for 15 min). The resulting cDNA products were diluted with 130 μL DEPC-treated water to achieve a final concentration of 3.3 ng/μL. Quantitative PCR reaction was prepared using SensiFAST SYBR® No-ROX kit (Bioline), cDNA product (3 μL of 3.3 ng/μL) and transcript-specific forward and reverse oligos (0.8 μL of 10 μM oligo per reaction). The housekeeping gene *GAPDH* was used as reference.

Quantitative PCR was then performed using an Applied Biosystems QuantStudio 5 Real-Time PCR system. All quantitative PCR reactions were performed in triplicates and repeated three times. Quantitative PCR data were first analyzed by correcting all the cycles threshold (Ct) values to that of Ct values of *GAPDH* of the parental_DMSO condition. The corrected Ct values for each gene in each condition were then compared to the Ct values of the respective gene in the parental_DMSO condition.

Flow cytometry

Cells were trypsinized and resuspended in FACS buffer (2% FBS, 1 mM EDTA in PBS). Cells were analyzed using a BD FACSCalibur or a BD LSRFortessa X-20 flow cytometer.

QUANTIFICATION AND STATISTICAL ANALYSIS

Western blot data was quantified using Image Studio software (Li-Cor) or ImageJ and graphs were plotted using Prism (GraphPad). Representative images of at least three independent experiments are shown. Error bars represent the standard deviation.

Molecular Cell, Volume *79*

Supplemental Information

Quality Control of ER Membrane Proteins

by the RNF185/Membralin Ubiquitin Ligase Complex

Michael L. van de Weijer, Logesvaran Krshnan, Sabrina Liberatori, Elena Navarro Guerrero, Jacob Robson-Tull, Lilli Hahn, Robert Jan Lebbink, Emmanuel J.H.J. Wiertz, Roman Fischer, Daniel Ebner, and Pedro Carvalho

Supplemental Figure S1

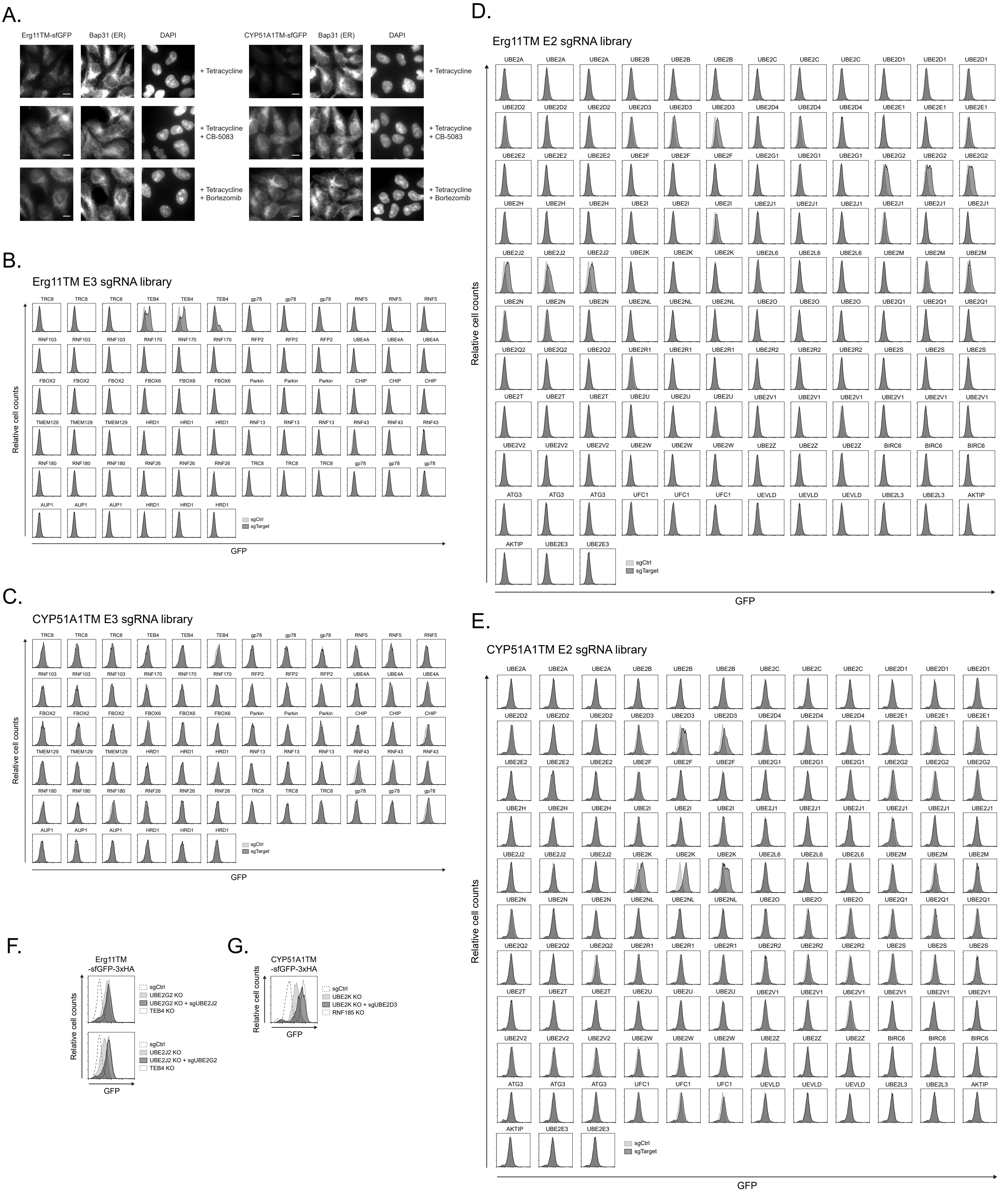

Figure S1. Focused CRISPR-Cas9 E3 and E2 library screens identify factors involved in the degradation of Erg11TM and CYP51A1TM, related to Figure 1.

(A) Erg11TM and CYP51A1TM localize to the ER. Flp-In T-REx 293 cells expressing Erg11TM or CYP51A1TM were incubated with either the p97 inhibitor CB-5083 (4 h; 2.5 µM) or the proteasome inhibitor bortezomib (4 h; 500 nM). Erg11TM and CYP51A1TM were detected by GFP fluorescence. BAP31 was used as an ER-marker and nuclei were stained with DAPI. Bars, 10 µm.

(B-E) CRISPR-Cas9 targeted screens of selected E3 and E2 libraries to identify factors involved in the degradation of Erg11TM and CYP51A1TM. Flp-In T-REx 293 cells expressing Erg11TM or CYP51A1TM were transfected with sgRNAs against indicated genes. Levels of Erg11TM and CYP51A1TM were analyzed by flow cytometry (based on GFP fluorescence).

(F-G) Redundancy between the E2 ubiquitin conjugating enzymes involved in the degradation of Erg11TM and CYP51A1TM. Flp-In T-REx 293 cells expressing (F) Erg11TM or (G) CYP51A1TM and deficient for a single E2 were additionally depleted for the indicated E2 enzyme. As a reference control, a cell line deficient for the E3 essential for the degradation of either Erg11TM or CYP51A1TM was included. Levels of Erg11TM and CYP51A1TM were analyzed by flow cytometry (based on GFP fluorescence).

Supplemental Figure S2

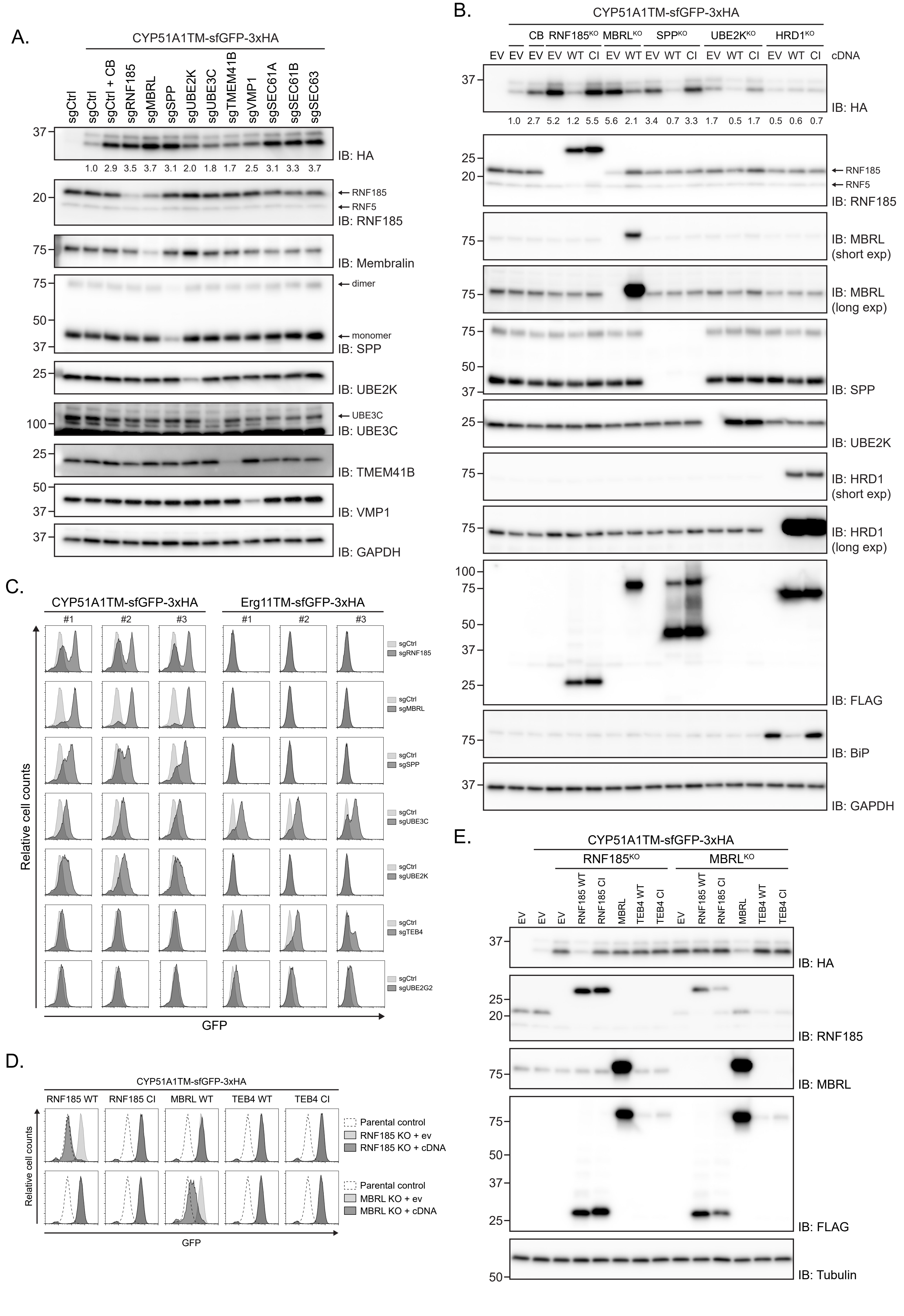

Figure S2: Additional validation of the hits identified in the CRISPR-Cas9 screen, related to Figure 2.

(A) Lysates of cells transfected with indicated sgRNAs were subjected to SDS-PAGE followed by immunoblotting. CB indicates cells treated with the p97 inhibitor CB-5083. Relative CYP51A1TM levels are displayed below the anti-HA blot, normalized to the GAPDH loading control.

(B) Lysates of clonal RNF185, MBRL, SPP, UBE2K, and HRD1 KO cells expressing cDNAs encoding either wildtype (WT), a catalytically inactive (CI) mutant, or an empty vector (EV) were subjected to SDS-PAGE followed by immunoblotting. Parental CYP51A1TM-expresing cells, either untreated or treated with the p97 inhibitor CB-5083 (CB), were used as control. Relative CYP51A1TM levels are displayed below the anti-HA blot, normalized to the GAPDH loading control.

(C) ERAD factor dependencies of CYP51A1TM and Erg11TM substrates. Flp-In T-REx 293 cells expressing CYP51A1TM or Erg11TM were transfected with sgRNAs targeting the indicated genes, or a control sgRNA (sgCtrl). Levels of Erg11TM and CYP51A1TM were analyzed by flow cytometry (based on GFP fluorescence).

(D,E) RNF185 and MBRL have unique, non-redundant roles in ERAD. CYP51A1TM levels were analyzed by (D) flow cytometry or immunoblotting (E) in clonal RNF185 and MBRL KO cells expressing cDNAs encoding either wild type RNF185 (RNF185 WT), a catalytically inactive RNF185 mutant (RNF185 CI), MBRL, TEB4 WT, TEB4 CI, or an empty vector (EV). In (E) Tubulin was used as a loading control and detected with an anti-Tubulin antibody.

Supplemental Figure S3

A.

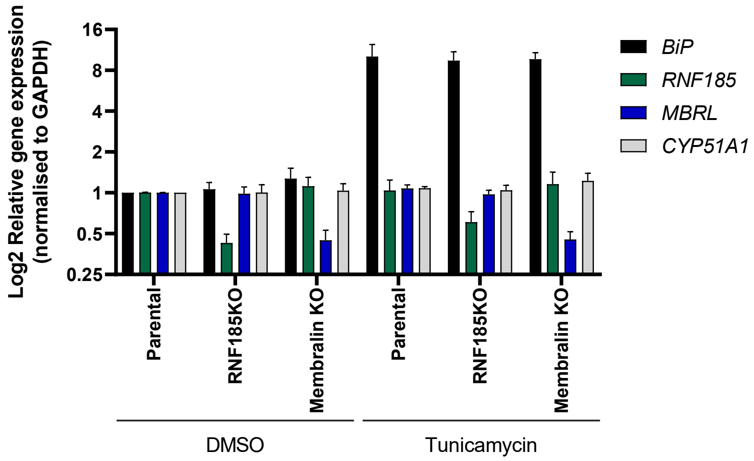

B.

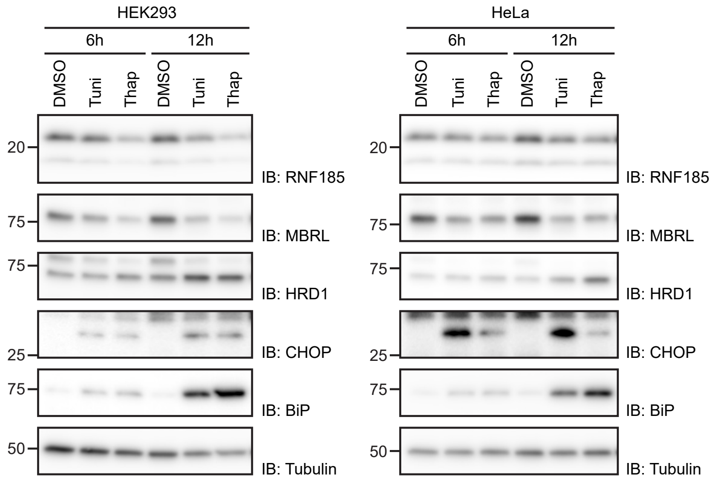

C.

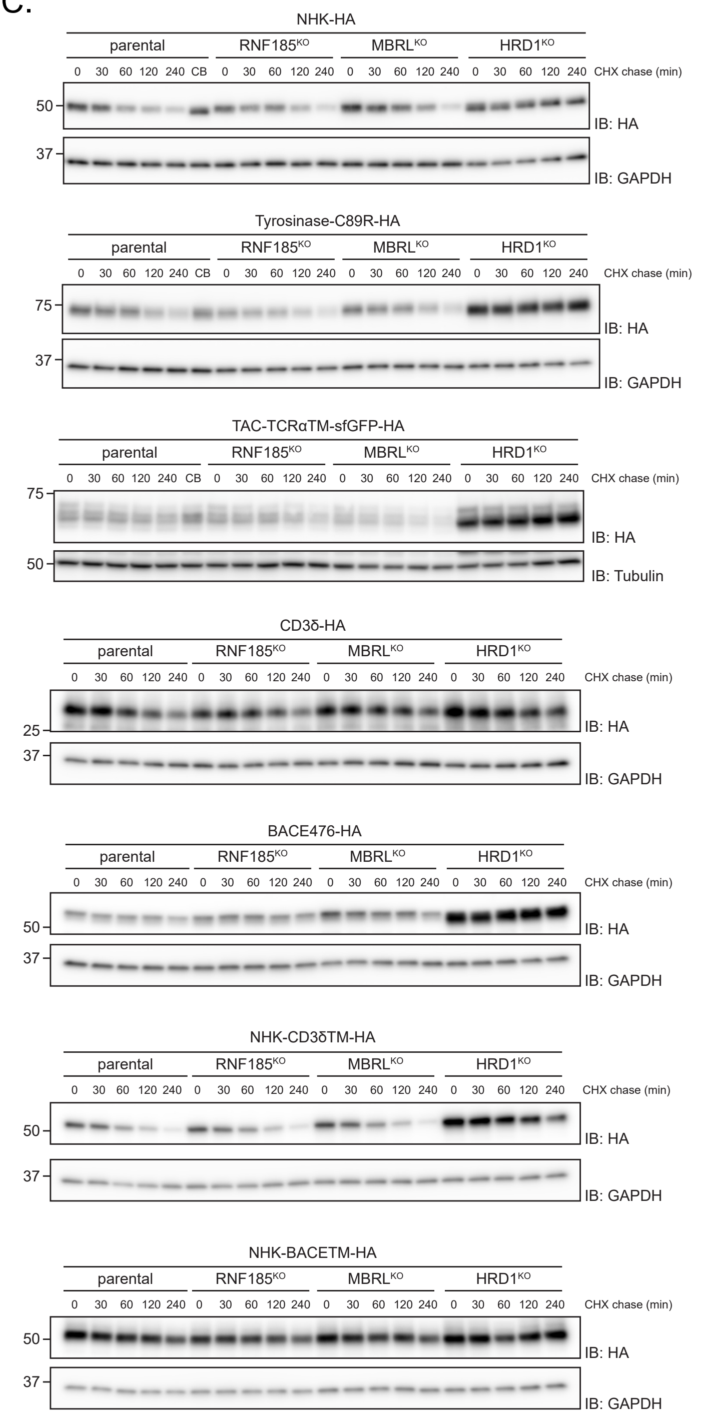

D.

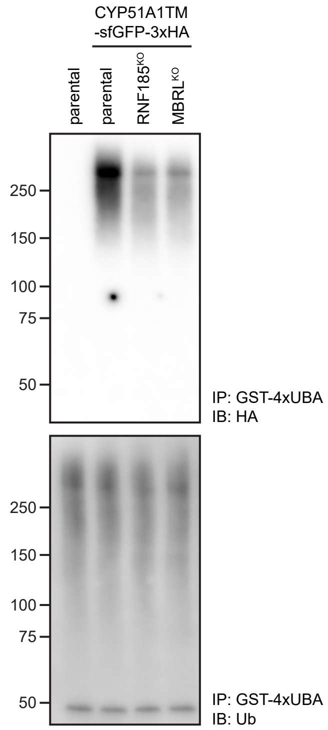

Figure S3: Loss of RNF185 and MBRL does not have a general effect on ER stress and ERAD, related to Figure 3.

(A) RNF185 and MBRL KO cells do not have significant UPR activation and are still capable of mounting an UPR response. T-Rex Flp-In HEK293 parental, RNF185 KO and Membralin KO cells were treated with either DMSO or 2 µg/mL Tunicamycin for 12 hours. Quantitative PCR was performed on *RNF185, TMEM259 (Membralin), CYP51A1* and *GRP78 (BiP)*. The house-keeping gene *GAPDH* was used as a reference. Reactions were performed with three independent biological samples, each analyzed in three technical replicates. Error bars represent the standard deviation.

(B) RNF185 and MBRL protein levels are not increased upon pharmacological UPR activation. HEK293 and HeLa cells were treated with either DMSO, 2 µg/mL Tunicamycin, or 1 mM Thapsigargin for 6 or 12 hours. Cell extracts were analyzed by SDS–PAGE and immunoblotted for the indicated proteins. Tubulin was used as a loading control and detected with an anti-Tubulin antibody.

(C) Degradation of the indicated canonical ERAD substrates was analyzed upon inhibition of protein synthesis with cycloheximide (CHX) in parental, RNF185, MBRL and HRD1 KO cells. Cell extracts were analyzed by SDS–PAGE and immunoblotting.

(D) Ubiquitination of CYP51A1TM is dependent on RNF185 and MBRL. Ubiquitin conjugates from parental, RNF185 KO and MBRL KO cells were isolated using GST-4xUBA. Eluted proteins were analyzed by SDS-PAGE followed by immunoblotting with anti-HA (top) and anti-Ubiquitin (bottom) antibodies. Parental cells not expressing CYP51A1TM were used as control.

Supplemental Figure S4

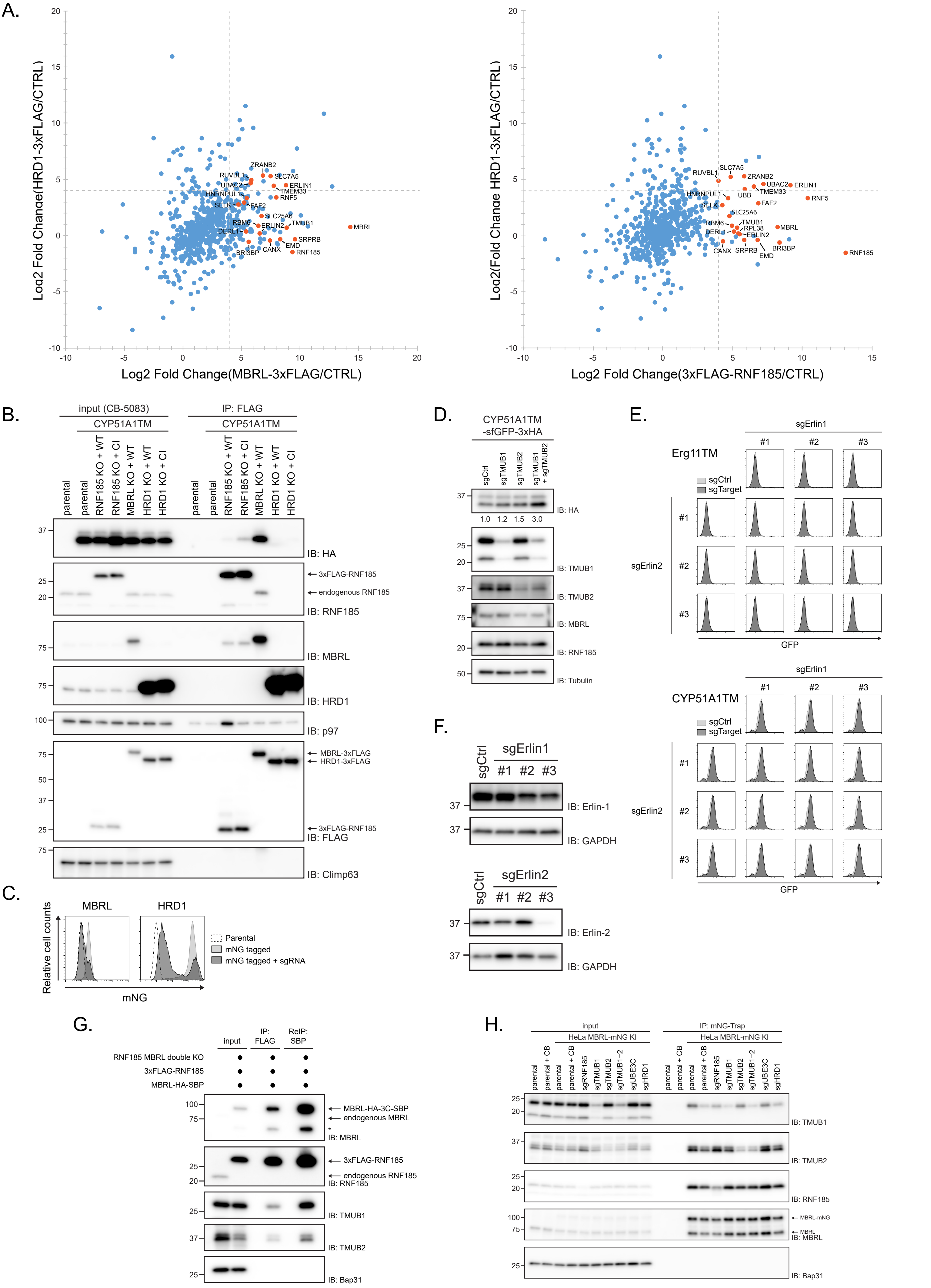

Figure S4: Analysis of the RNF185-Membralin complex, related to Figure 4.

(A) Proteins co-precipitating with MBRL-3xFLAG (x-axis; left graph) or 3xFLAG-RNF185 (x-axis; right graph) versus HRD1-3xFLAG/ctrl (y-axis; both graphs) as analyzed by mass spectrometry. The enrichment of the proteins associated to FLAG-tagged baits over an untagged control was used to calculate the log2 fold changes. The cutoff enrichment was arbitrarily set to 4 (dashed lines). Proteins enriched above the cutoff in both MBRL and RNF185 immunoprecipitates from Figure 4A are annotated and orange colored.

(B) Analysis of proteins co-precipitating with FLAG-tagged RNF185, MBRL and HRD1 by immunoblotting. Parental control cells and the indicated KO cells expressing the corresponding FLAG-tagged proteins treated with the p97 inhibitor CB-5083 (4 h) were lysed and detergent extracts (1% DMNG) subjected to immunoprecipitation with anti-FLAG resin. Eluted proteins were analyzed by SDS-PAGE and immunoblotting. WT, wildtype; CI, catalytically inactive.

(C) Flow cytometric validation of endogenously mNG-tagged MBRL and HRD1. Cells were transfected with sgRNA targeting MBRL or HRD1, after which mNG fluorescence was assessed using flow cytometry. mNeonGreen-negative cells (parental) were included as a baseline control.

(D) Western blot validation of sgRNA-mediated depletion of TMUB1 and TMUB2. Relative CYP51A1TM levels are displayed below the anti-HA blot, normalized to the Tubulin loading control.

(E) Erlin-1 and Erlin-2 are not functionally involved in the degradation of Erg11TM and CYP51A1TM. Cells were depleted for either Erlin-1, Erlin-2, or both using sgRNAs. Levels of CYP51A1TM-sfGFP-3xHA were analyzed by flow cytometry (based on GFP fluorescence).

(F) Western blot validation of sgRNA-mediated depletion of Erlin-1 and Erlin-2.

(G) RNF185, MBRL and TMUB1/2 assemble in a defined biochemical complex, as assessed by sequential immunoprecipitation. Extracts from cells expressing 3xFLAG-RNF185 and MBRL-HA-SBP were immunoprecipitated with anti-FLAG resin followed by native FLAG-peptide elution. Eluates were re-immunoprecipitated with Streptavidin beads. Final elutions were analyzed by SDS-PAGE and immunoblotting with the antibodies directed to the indicated proteins. The asterisk (*) indicates a truncated MBRL product.

(H) RNF185-MBRL complex composition. Endogenous MBRL tagged with mNG on the C-terminus was immunoprecipitated from 1% DMNG lysates of the indicated cell lines. Eluted proteins were analyzed by immunoblotting.

Supplemental Figure S5

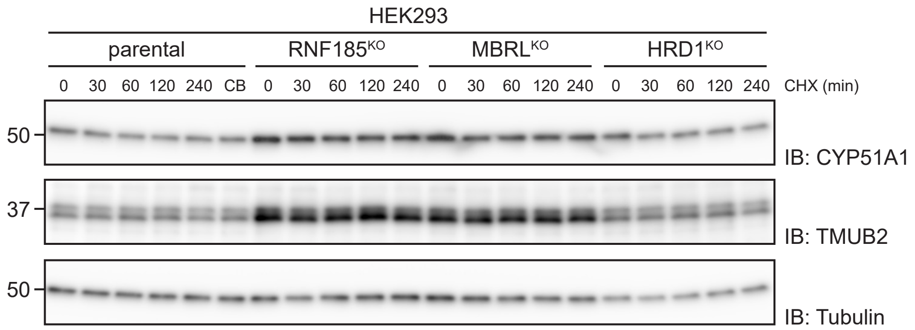

Figure S5: Turnover of CYP51A1 and TMUB2 in RNF185 and MBRL KO cells, related to Figure 5.

The turnover of endogenous CYP51A1 and TMUB2 was assessed by inhibition of protein synthesis using cycloheximide (CHX) for indicated time points in parental, RNF185, MBRL and HRD1 KO cells. Lysates were subjected to SDS-PAGE followed by immunoblotting for the indicated proteins. CB, CB-5083-treated.

Supplemental Figure S6

A.

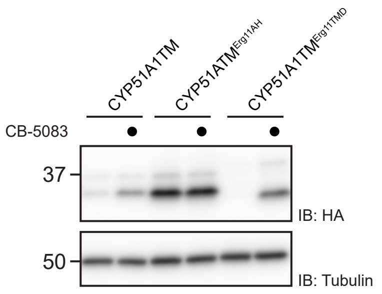

B.

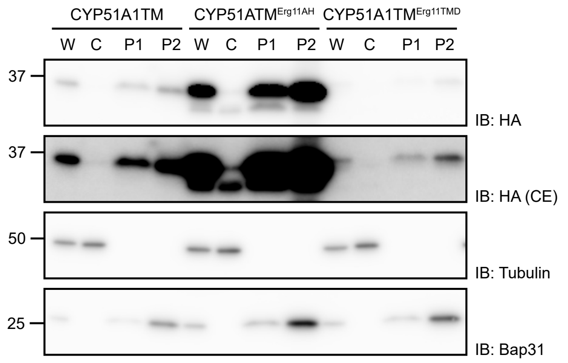

C. CYP51A1TM^Erg11TMD-sfGFP-3xHA

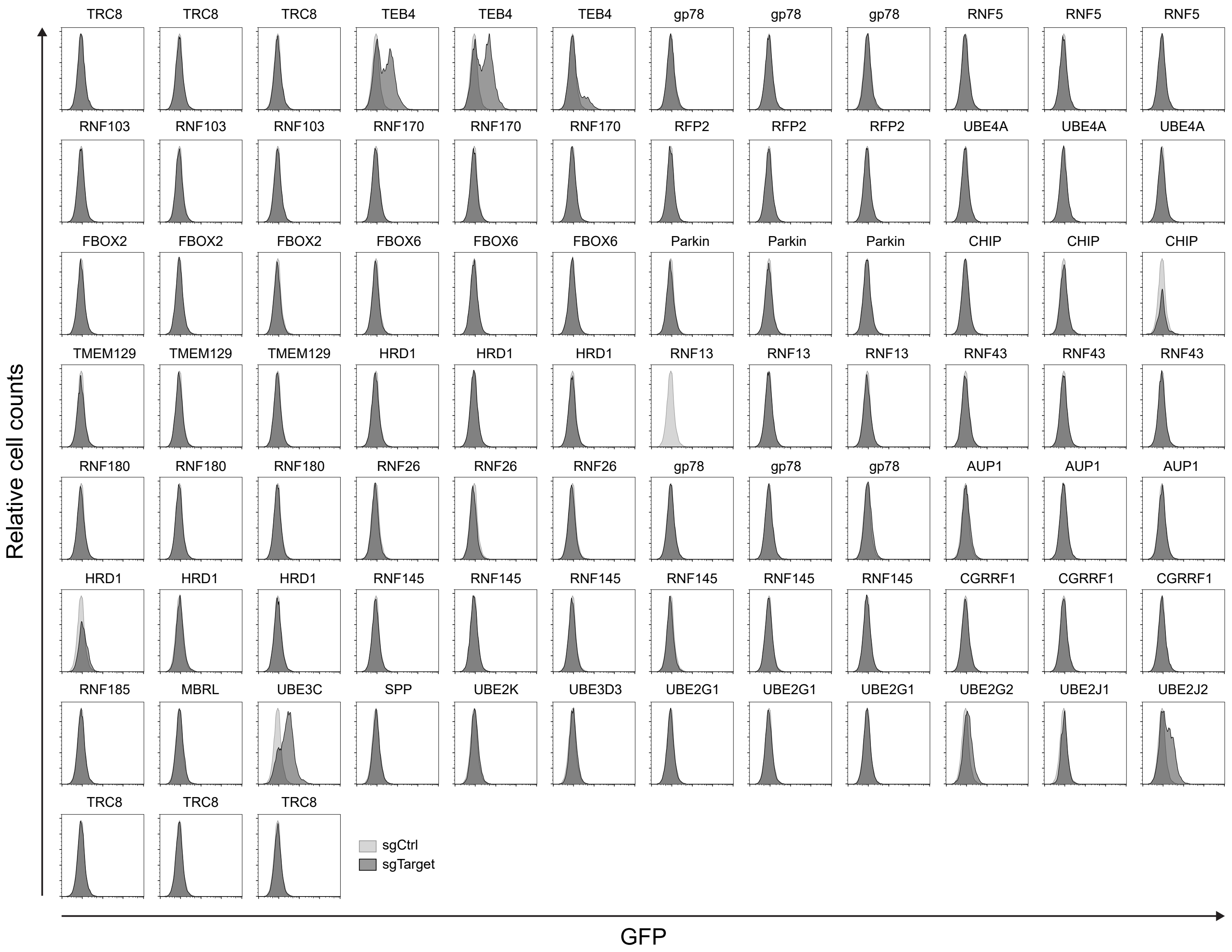

Figure S6: Analysis of CYP51A1TM chimeric constructs, related to Figure 6.

(A) CYP51A1TM and chimeric constructs were expressed in Flp-In T-REx HEK293 cells, after which cells were cultured in the absence or presence of the p97 inhibitor CB-5083 (4 h). Protein levels were assessed by immunoblotting. Tubulin was used as loading control.

(B) CYP51A1TM and its chimeric derivatives are stably associated with membranes, as determined by subcellular fractionation. Postnuclear supernatants (W) were fractionated into a cytosolic soluble fraction (C) and a crude membrane pellet (P1). Membranes in P1 were salt washed and carbonate-treated (P2) to remove all peripherally associated proteins. Fractions were subjected to SDS-PAGE and western blotting analysis. The partitioning of the HA-tagged substrates was compared to endogenous soluble (Tubulin) and integral membrane (BAP31) proteins.

(C) Flp-In T-REx 293 cells expressing CYP51A1TM^{Erg11TMD}-sfGFP-3xHA were transfected with sgRNAs against indicated targets. Levels of CYP51A1TM^{Erg11TMD}-sfGFP-3xHA were analyzed by flow cytometry (based on GFP fluorescence).
